# Genetic Epidemiology of Bovine Leptospirosis: A Global Perspective from Sequence and Genome Datasets

**DOI:** 10.3390/ani16132017

**Published:** 2026-07-02

**Authors:** Luiza Aymée, Ana Luiza dos Santos Baptista Borges, Maria Isabel Nogueira Di Azevedo, Walter Lilenbaum

**Affiliations:** 1 Laboratory of Veterinary Bacteriology, Biomedical Institute, Federal Fluminense University, Rio de Janeiro 24210-130, Brazil; 2 Department of Microbiology, Immunology and Parasitology, Faculty of Medical Sciences, Rio de Janeiro State University, Rio de Janeiro 20550-170, Brazil; 3 Laboratory of Investigation in Medical Microbiology, Paulo de Góes Institute of Microbiology, Federal University of Rio de Janeiro, Rio de Janeiro 21941-901, Brazil

**Keywords:** cattle, climate, genotyping, genetic markers, *Leptospira*, WGS

## Abstract

Leptospirosis is an important reproductive disease in bovine hosts, yet its epidemiology is still largely inferred from serology, which provides limited resolution in bovines. Although genetic studies based on genotyping and whole-genome sequencing approaches are increasing, a comprehensive overview is still lacking. We analyzed bovine-origin sequences and genome metadata from databases to characterize patterns of *Leptospira* species and serogroups and to describe genotyping methods and molecular markers. Metadata were retrieved from GenBank and the Institut Pasteur cgMLST databases until January 2026. Extracted variables included species, genotyping approach/markers, serological classification of isolates, sample type and origin (renal vs. genital), clinical signs, and geographic location; climates were assigned using Köppen–Geiger classification. After selection, 569 records were retrieved: 411 single-locus sequences (eight molecular markers), 95 MLST profiles, and 63 genomes, from 35 countries, with most reports from South America (57.6%). Records spanned tropical, temperate, steppe, Mediterranean, and continental-cold climates. Nine species and 14 serogroups were identified; tropical and temperate areas showed greater diversity. The main agents, *L. interrogans*, *L. borgpetersenii*, and serogroup Sejroe, were predominant and widespread. Notably, *L. noguchii*, *L. santarosai, L. venezuelensis*, and *L. wolffii* were mostly concentrated in the Americas. Most records were from renal samples (68.7%), indicating limited focus on genital leptospirosis, and 83.5% lacked clinical information, limiting links between strains and manifestations. Overall, bovine leptospirosis is worldwide distributed, but gaps persist in marker standardization for single-locus sequencing and genome availability. Standardized genotyping, increasing genital sampling, and improving clinical metadata are essential for refining surveillance and clarifying the role of emerging species.

## Simple Summary

Bovine leptospirosis is an important reproductive disease worldwide, but its genetic epidemiology remains poorly explored. Understanding the global distribution of leptospiral species and the methods used for their detection and characterization is essential to better estimate the burden of the disease, particularly given the international trade of cattle, reproductive bioproducts, and the widespread sharing of leptospiral vaccines. In this study, we evaluated sequence and genome records of bovine leptospires and their associated metadata to identify patterns related to species distribution, countries, climate regions, sample origin, and clinical information. Our findings showed that bovine leptospiral records have been reported across all inhabited continents and in five climate types, indicating that the disease is not restricted to tropical regions but also occurs in dry climates. Nine *Leptospira* species were identified, including emerging species beyond the classical *L. interrogans* and *L. borgpetersenii*. Multiple serogroups were also inferred from isolates, highlighting the role of non-Sejroe leptospires in cattle infections. This study also revealed important gaps, including the limited number of published bovine isolates and genomes, the predominance of records from renal-origin samples, the scarcity of data from genital and other anatomical sites, and the frequent absence of clinical information in available metadata.

## 1. Introduction

Leptospirosis is a neglected zoonotic disease with worldwide distribution, considered endemic in tropical regions and reemerging in temperate settings [1]. This disease is a significant concern for livestock, causing substantial economic losses, particularly in cattle [2,3]. Spirochetes of the genus *Leptospira* are the etiological agents, which can be classified using both genetic and serological (phenotypic) approaches. Genetically, 74 *Leptospira* species have been described to date and are divided into pathogenic subclades P1 and P2 (formerly “intermediate” species) and saprophytic subclades S1 and S2 [4]. In parallel, serological classification is based on the antigenic composition of outer-membrane lipopolysaccharides (LPS), dividing leptospires into more than 300 serovars, which are grouped into 26 serogroups based on LPS similarity [5]. Serovar identification can help determine whether a strain is host-adapted or incidental to a given animal species, providing important insights into the disease’s epidemiology [6]. Because genetic and serological classification systems do not converge, they should be used simultaneously whenever possible.

Leptospires can infect a wide range of domestic and wild animals besides humans [2,7]. In cattle, leptospirosis is recognized as one of the most relevant infectious causes of reproductive failures, leading to embryonic death and repeat breeding, as well as abortion at any stage of gestation, stillbirth, or the birth of weak calves [8,9]. Notably, reproductive disease has frequently been linked to the Sejroe serogroup, which is considered host-adapted to cattle [10]. However, incidental strains such as Icterohaemorrhagiae, Grippotyphosa, Pomona, and others, which are typically host-adapted to different animal species, have also been reported to occur in bovine abortion outbreaks [9,11].

Transmission of leptospires to cattle is related to cow-to-cow spread of strains through contact with contaminated urine and the environment [12]. It is also suggested to occur through the venereal route, including natural mating and the use of contaminated semen or other reproductive biological materials (e.g., embryos) [9,13]. Cattle may become infected by incidental strains from contaminated environments shared with other animal reservoirs [6]. After infection, leptospires colonize the renal tract and are shed in cattle urine, a phenomenon described for decades [12]. Reproductive tract colonization has been described in the 1980s [14], but gained more relevance recently, as it was associated with Bovine Genital Leptospirosis [15]. Despite colonization patterns, effective control of leptospirosis relies on appropriate vaccines, environmental management, and antimicrobial strategies [9]. In this context, understanding which strains are circulating and their infection patterns is essential to refine vaccine composition and support the development of new formulations [16]. A wide epidemiological panorama is particularly important given the international sharing of commercial vaccines and the trade of live animals and reproductive byproducts such as semen.

Historically, most of the epidemiological evidence on bovine leptospirosis has come from serological surveys using the Microscopic Agglutination Test (MAT), recommended by the World Organisation for Animal Health and applied worldwide [17]. Nevertheless, serology has important limitations: it primarily indicates exposure rather than active infection/shedding, cannot reliably distinguish infection-induced antibodies from vaccine-induced antibodies, and can cause cross-reactivity across serogroups [18], jeopardizing epidemiological studies. Particularly in bovines, MAT may show reduced sensitivity because cattle frequently mount weak humoral responses to *Leptospira* spp. [19]. In parallel to serology, efforts to obtain isolates have been used in the past to support epidemiological inferences. However, since leptospiral culturing remains challenging, culture-independent approaches have been adopted, including direct sequencing from clinical samples to characterize circulating species and infection patterns [18]. Although these genetic approaches have been applied in many local studies, there is still no standardization of the genetic markers applied [20].

Given persistent knowledge gaps and heterogeneous methodologies, a comprehensive overview of the genetic aspects of bovine leptospirosis is still lacking. Due to the global trade of live bovines or their reproductive byproducts, as well as their growing impact in the One Health scenario, there is a current need to strengthen our understanding of its epidemiology. In this context, this study aimed to analyze bovine-origin *Leptospira* genomes and sequences with associated public metadata to assess species and serogroup distribution across continents and climates, as well as genotyping methods, sample types, and clinical data.

## 2. Materials and Methods

Metadata records, defined as information associated with sequence and genome entries deposited in public databases, were retrieved from sequence and genome deposited in NCBI GenBank (NIH) and from the Institut Pasteur *Leptospira* cgMLST BIGSdb database (https://bigsdb.pasteur.fr/cgi-bin/bigsdb/bigsdb.pl?db=pubmlst_leptospira_isolates accessed on 28–30 January 2026). Data retrieval and curation were performed between 7 January and 30 January 2026. In GenBank, records were identified using the following keyword combinations: “*Leptospira* AND bovine”, “*Leptospira* AND cattle”, “*Leptospira* AND Bos taurus”, “*Leptospira* AND *Bos indicus*”, “*Leptospira* AND cow”, and “*Leptospira* AND bull.” In the Pasteur BIGSdb database, isolates were filtered using the Host = bovine field. Duplicate records were excluded, including identical sequences deposited more than once, metadata entries corresponding to equal sequences from the same samples, and contigs derived from the same genome. For strains with both single-locus sequences and subsequently available genome data, only the genome record was considered. Entries corresponding to non-*Leptospira* bacteria or non-bovine hosts were also excluded.

From that sequences/genomes, the following data were extracted: (a) the *Leptospira* species identified; (b) the genetic typing approach (e.g., Whole-Genome Sequencing, Multi-Locus Sequence Typing, or single-locus/single-marker sequencing) and the target genetic markers of each record; (c) sample type (clinical specimen vs. isolate); (d) isolate serological (phenotypic) characterization (when available); (e) sample origin (e.g., renal vs. genital/reproductive tract); (f) reported clinical signs (when available); and (g) geographic information (state, country, and continent). Information was retrieved directly from database fields and, when missing or incomplete, was complemented using the associated publication. Retrieved records and metadata are provided in Supplementary Table S1.

Temporal analysis was performed based on the dates of sequence or isolate recovery. Records with available dates were grouped into 10-year eras, from the earliest available record to 2025. For each era, the number of sequence entries, the methodology used, the most frequent genetic markers, the most frequent leptospiral species, and the countries of origin were evaluated. A temporal timeline figure was generated using NotebookLM and subsequently modified in Adobe Photoshop.

**Figure 1:**
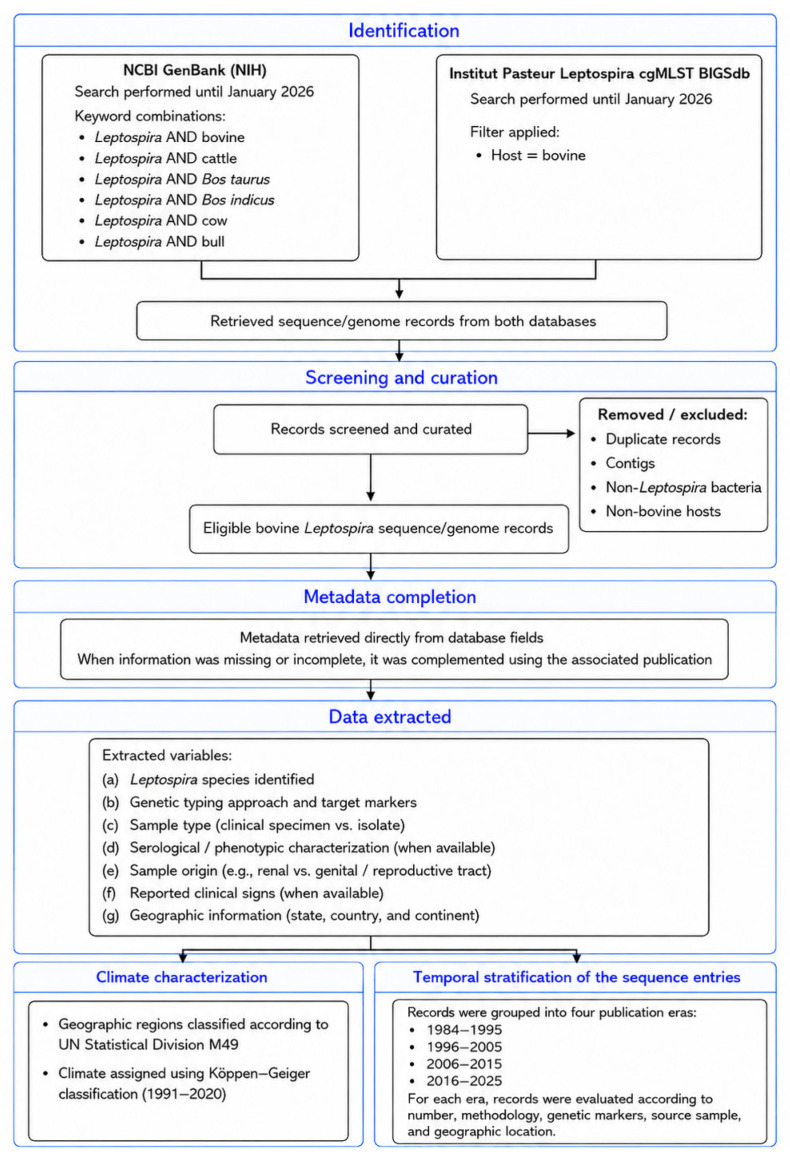
Workflow of retrieval and curation of metadata records from bovine *Leptospira* sequences and genomes deposited in public databases.

Geographic regions were first classified according to the United Nations M49 standard classification from the UN Statistics Division (https://unstats.un.org/unsd/methodology/m49/, accessed on 10 February 2026). Climate categories were then assigned to each location using the 1991–2020 Köppen–Geiger classification based on the University of Vienna (VU Wien) Google Earth dataset (https://koeppen-geiger.vu-wien.ac.at/present.htm, accessed on 10 February 2026), following Rubel et al. [21]. For records with state and country information, climate was assigned at the state/locality level. For records lacking state-level information, the predominant national climate class was assigned when the country or territory had an area of up to 550,000 km^2^ and a single Köppen–Geiger climate class covering more than 70% of its land area. To simplify the analysis, Köppen–Geiger categories were grouped into the major climate classes. The map, charts, and heatmap depicting leptospiral species distribution across geographic regions and climates were generated in R (v4.5.2) using the ggplot2 and rnaturalearth packages. The workflow of this study is described in the flowchart shown in Figure 1.

Statistical analyses were performed in R (v4.5.2). To evaluate the methodological resolution supporting species-level identification, records were classified according to the typing approach used and assigned an ordinal methodological weight: whole-genome sequencing/cgMLST = 3, MLST = 2, and single-locus sequence analysis = 1. Fisher’s exact test was used to assess whether *Leptospira* species were identified in similar proportions across methodological categories. In addition, methodological weights were compared among species using the Kruskal–Wallis test to evaluate whether methodological resolution differed among species.

The distribution of leptospiral species across the main climate classes was also assessed using Fisher’s exact test, excluding records without species-level identification or climate classification. The same approach was applied to evaluate the distribution of Sejroe versus non-Sejroe serogroups across climate classes. Standardized residuals were calculated descriptively to identify species–climate and serogroup–climate combinations contributing most to significant associations. Positive residuals indicated higher-than-expected occurrence, whereas negative residuals indicated lower-than-expected occurrence. Residuals ≥ 2 or ≤−2 were considered relevant deviations from the expected distribution.

Finally, chi-square goodness-of-fit tests were used to assess whether sample types and clinical data availability were unevenly distributed in the dataset. For clinical information, records were classified as either associated with clinical data or not. Records from apparently asymptomatic animals sampled at slaughterhouses without reproductive history, together with records for which clinical data were not reported, were considered not associated with clinical information. Statistical significance was set at *p* < 0.05.

## 3. Results

A total of 15,937 sequences/genomes were retrieved from GenBank using the predefined queries, and 108 bovine isolates were obtained from the Institut Pasteur *Leptospira* BIGSdb. After applying the selection criteria, 569 metadata records were included (523 from GenBank and 46 from Pasteur BIGSdb).

The characterization of the records revealed *Leptospira interrogans* (208/569 records, 36.5%), *L. borgpetersenii* (194/569, 34.1%), *L. santarosai* (49/569, 8.6%), *L. kirchneri* (36/569, 6.3%), *L. noguchii* (28/569, 4.9%), *L. venezuelensis* (4/569, 0.7%), *L. wolffii* (3/569, 0.5%), *L. weilli* (1/569, 0.2%), and *L. alexanderi* (1/569, 0.2%). Additionally, 45 (7.9%) records were only characterized at the genus level. Regarding the genotyping method, 411 of the records corresponded to single-locus sequences, 95 to MLST profiles, and 63 to whole-genome data. The genetic markers used for single-locus sequences were *secY*, *rrs* (16S rRNA), *lfb1*, *rpoB*, *flaB*, *lipL32*, *gmlU*, and *gyrB*. The proportion of genomes, single-locus markers, and MLST schemes is described in Table 1. Among the included records, 314 (55.2%) were generated directly from clinical samples (direct genotyping), whereas 255 (44.8%) were obtained from cultured isolates. Serological characterization was available for 208 of the 255 isolates. Among these, 84 (40.4%) belonged to the Sejroe serogroup, whereas 120 (57.7%) were classified as non-Sejroe, including Pomona, Icterohaemorrhagiae, Grippotyphosa, Autumnalis, Australis, Panama, Pyrogenes, Tarassovi, Bataviae, Mini, Sarmin and Hebdomadis.

To evaluate species identification according to methodological resolution, we compared the distribution of *Leptospira* species across methodological categories that differed in robustness and taxonomic resolution. Fisher’s exact test with simulated *p*-values showed a significant association between species and methodological category (*p* < 0.001), indicating that species were not identified in similar proportions across single-locus sequence analysis (SLS), MLST, and WGS/cgMLST approaches. The mean methodological weight also varied among species, with higher weights reflecting a greater contribution of high-resolution methods, such as WGS and cgMLST. Among species represented by larger sample sizes, *L. noguchii* showed the highest mean methodological weight, followed by *L. santarosai*. In contrast, *L. interrogans*, *L. borgpetersenii*, *L. kirschnerii* exhibited lower mean weights, indicating a greater reliance on single-locus sequence data (Table 2). Kruskal–Wallis test confirmed significant differences in methodological weight among species (*p* < 0.0001).

**Figure 2:**
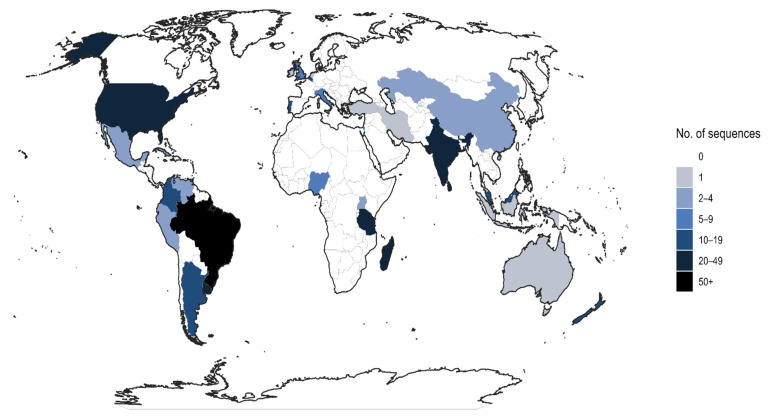
Frequency of available *Leptospira* spp. bovine sequences and genomes by country of origin.

**Figure 3:**
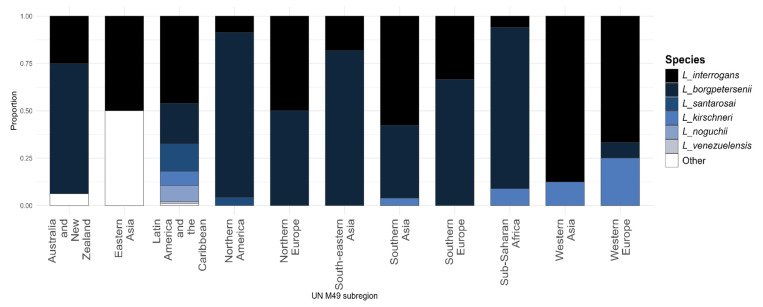
Distribution of leptospiral species identified in bovines across geographic subregions, according to the UN M49 classification.

The records were retrieved from 35 countries on five continents (Africa, America, Asia, Europe, and Oceania). The proportions of sequences by continent are shown in Table 3, while the frequencies of records by country are mapped in Figure 2 and shown in Supplementary Table S2. The distribution of leptospiral species by geographic regions is demonstrated in Figure 3 and in Supplementary Table S2. Given the potential identification bias among sequences from South America, records from this continent were analyzed separately in Supplementary Table S3. Compared with the rest of the world, South America accounted for most *secY* sequences (75.7% of the total), most *L. interrogans* identifications (70.7% of the total), and the highest proportion of genital samples (95.3%). Additionally, non-Sejroe isolates were more frequent than Sejroe isolates among climate-associated records (65.7% vs. 34.3%, respectively).

Köppen–Geiger climate classes were assigned to 468 records. Exact location data, including state and country, was available for 431 records. For the remaining 37 records, state-level information was unavailable; however, these records originated from countries or territories smaller than 550,000 km^2^ and characterized by a predominant climate class, namely Cuba, Israel, Italy, France, La Réunion, New Zealand, and Uruguay. Eleven Köppen–Geiger classes were identified (Table 3) and subsequently grouped into five main climate categories: Tropical (303/468 records, 64.7%), Temperate (135/468, 28.8%), Mediterranean (15/468, 3.2%), Continental-cold (12/468, 2.6%), and Steppe (3/468, 0.6%).

Leptospiral species were not equally distributed across climate zones (*p* < 0.001). Tropical and temperate climates accounted for the highest number of reports. In tropical settings, five identified species were reported, mainly *L. interrogans* (34.0%), *L. borgpetersenii* (29.7%), *L. santarosai* (13.5%), *L. kirschneri* (8.3%), and *L. noguchii* (5.6%). In temperate settings, seven species were identified, with predominance of *L. interrogans* (48.1%) and *L. borgpetersenii* (35.6%), followed by *L. noguchii* (7.4%), *L. wolffii* (2.2%), *L. kirschneri* (2.2%), *L. santarosai* (1.5%), and *L. alexanderi* (0.7%). Pairwise comparison confirmed that species distribution differed significantly between tropical and temperate climates (*p* = 0.0001). The distribution of Sejroe and non-Sejroe strains also differed significantly between tropical and temperate climates according to Fisher’s exact test (*p* = 0.043). Sejroe strains were overrepresented in temperate settings (standardized residual > 2), whereas non-Sejroe strains were overrepresented in tropical climates (standardized residual > 2).

**Figure 4:**
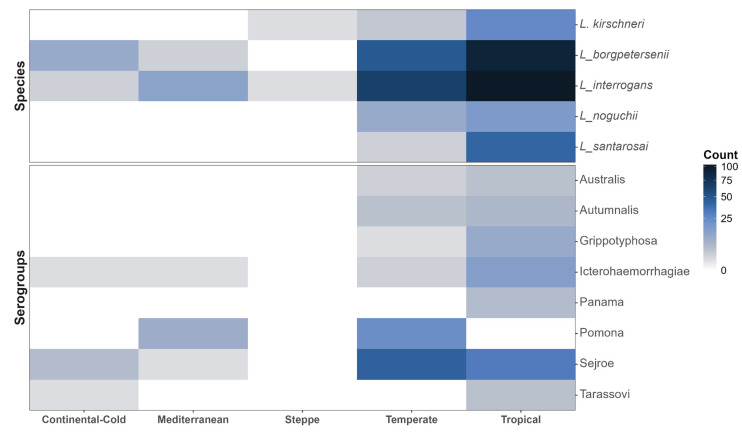
Distribution of *Leptospira* spp. species and serogroups across the main climate groups most frequently represented in the retrieved sequences. No serogrouped isolates were reported in records from the Steppe climate.

The distribution of the main leptospiral species and serogroups across climate categories is shown in Figure 4.

**Figure 5:**
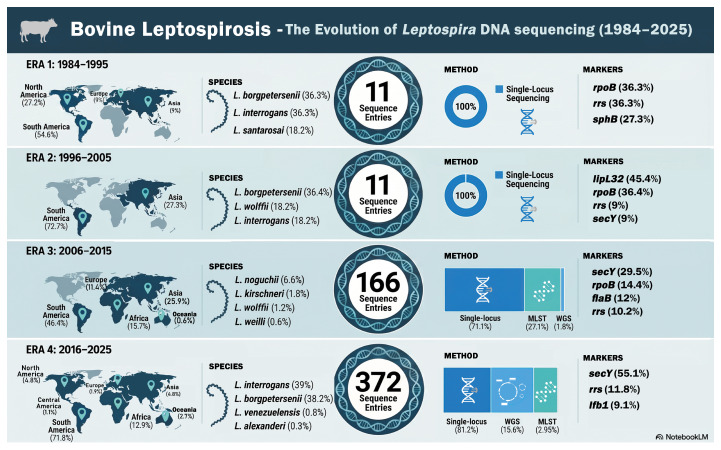
Temporal analysis and evolution of DNA sequencing records of bovine-origin *Leptospira*.

Temporal analysis was performed on 525 records that had available recovery dates, covering entries from 1984 to 2025. The main information for each era is summarized in Figure 5, and detailed data are provided in Supplementary Table S3. 

Finally, regarding host-related information (sample type/tissue system and clinical presentation), 391/569 (68.7%) records originated from the renal tract (urine or kidney), 86/569 (15.1%) from the genital tract (cervicovaginal mucus, uterine fragments, follicular fluid, semen, epididymis), 23/569 (4%) from blood or serum, and 18/569 (3.2%) from fetal tissues and/or placenta; for 51 records, the sample type was not reported. Sample types were significantly unevenly distributed, with renal tract samples predominating over the other categories (*p* < 0.001).

Clinical information was available for a subset of records: 76/569 (13.3%) were associated with reproductive failure, 13/569 (2.3%) from aborted fetuses, and five with systemic signs (e.g., pyrexia and hepatic failure). Most records (306/569, 53.8%) were obtained from apparently asymptomatic animals sampled at slaughterhouses, without reproductive history available, while clinical data were not reported for 169/569 (29.7%) records. In total, 475/569 records (83.5%) were not associated with any clinical data, which were significantly overrepresented in the database (*p* < 0.001).

## 4. Discussion

The present study addressed the global deposition of *Leptospira* sequences and genomes obtained from bovines. Nine leptospiral species were reported infecting cattle: seven from the P1 subclade, which includes the main pathogenic leptospires (*L. interrogans*, *L. borgpetersenii*, *L. kirschneri*, *L. santarosai, L. noguchii*, *L. weilli*, and *L. alexanderi*), and two from the P2 (intermediate) subclade (*L. venezuelensis* and *L. wolffii*). Not surprisingly, *L. interrogans* and *L. borgpetersenii* were the most frequently reported species, which is consistent with their recognized role as the main agents of bovine leptospirosis worldwide, particularly the genotypes of serovar Hardjo: Hardjoprajitno (*L. interrogans*), and Hardjobovis (*L. borgpetersenii*) [9,22]. It is noteworthy that both species were detected across the five continents (the Americas, Africa, Asia, Europe, and Oceania) at similar proportions, with Africa as the main exception, where *L. borgpetersenii* was slightly more frequent than *L. interrogans*. This pattern of distribution challenges a long-standing paradigm in the bovine leptospirosis literature, which suggests that *L. interrogans* predominates in Europe, whereas *L. borgpetersenii* is more common in other continents [23]. However, although these species were the most frequently identified in the databases, they were mainly represented by single-locus sequences rather than whole-genome records. For this reason, these records were assigned a lower weight due to their more limited genetic information. The scarcity of genomes available for these species, compared with single-locus sequences, may be related to the highly fastidious nature of the Hardjo genotypes, which are especially difficult to culture, even when compared to other leptospires [6,9,15].

Other leptospiral species are also concerning, especially because of the limited information on their roles as agents of bovine leptospirosis. *L. kirschneri* was also reported across six continents, except Oceania. Although it is less frequently reported in cattle, this species has been associated with acute, fatal disease in calves [24] and has also been described in adult bovines without systemic signs [25]. *L. santarosai* and *L. noguchii* are recognized as emerging pathogens and, in the last few years, have often been reported infecting cattle [26,27]. However, the available reports in bovines are currently restricted to the Americas. *L. santarosai* has been associated with the etiology of the syndrome Bovine Genital Leptospirosis (BGL), causing subclinical reproductive signs [15]. Although less frequently, *L. noguchii* has also been associated with reproductive disease, including abortion outbreaks, and it is a concerning agent from a One Health perspective [22,28]. In addition, the “intermediate” species *L. venezuelensis* and *L. wolffii* have also been reported only in the Americas, and little is known about their pathogenicity and association with clinical manifestations in bovines, despite evidence that they can cause clinical illness in humans [29]. Further studies are needed to clarify the roles of these emerging pathogenic leptospires and intermediate species in bovine leptospirosis, not only to understand their sanitary impact on cattle production better, but also to evaluate their ecological role and maintenance in the Americas.

Records of *Leptospira* sequences from bovines were identified in countries worldwide, reflecting the global distribution of the disease. Notably, most records originated from the Americas, particularly South America, which accounted for more than half of all reports (57.6%). This pattern is unsurprising given the presence of major cattle-producing countries in South America, such as Brazil, Uruguay, and Argentina, as well as the predominance of tropical settings characterized by high biodiversity, diverse reservoirs, and a wide range of biomes, including highly biodiverse ecosystems such as the Amazon and Atlantic forests [7]. However, the large number of records from South America should be interpreted cautiously, as it may have influenced some of the metrics observed in this study. This is particularly relevant for the high proportion of *secY* sequences and the greater number of records attributed to *L. interrogans*, which were, overall, the main genetic marker and species identified in this study. South America also accounted for nearly half of the serogrouped isolates, most of which were characterized as non-Sejroe, contrasting with the pattern observed in other regions. This finding may be associated with the high biodiversity and complex ecological environments, combined with the wide extent of extensive cattle farming systems. It is important to highlight that the number of records in a given region, whether high or low, should not be directly extrapolated as an indicator of leptospirosis impact or prevalence, since cattle population size, diagnostic capacity, and surveillance efforts can vary substantially among regions. This may be attributed, at least in part, to the silent and chronic condition of bovine leptospirosis, as well as clinical manifestations that may go unnoticed in the field despite causing economic losses, which may reflect in underdiagnosis [30].

Likewise, leptospiral records from bovines were reported across five climate groups: tropical, temperate, steppe, Mediterranean, and continental-cold. Tropical and temperate settings showed the greatest diversity of both leptospiral species and serogroups, suggesting a broader range of reservoirs. This pattern was not surprising, as leptospirosis has been for a long time recognized as endemic in tropical areas [1] but has been increasingly reported in temperate regions [31]. Interestingly, species distribution differed significantly between tropical and temperate climates. Although tropical regions yielded a higher number of records, temperate regions showed greater species diversity. *L. interrogans* and *L. borgpetersenii* predominated in both climate groups; however, tropical records showed a more balanced distribution among species. Taken together, these findings highlight the need for further studies on bovine leptospirosis in temperate areas, where the observed variability remains insufficiently understood from an epidemiological perspective.

In this study, we applied the Köppen–Geiger classification, which categorizes regions based on major climate types, precipitation, and temperature [32]. Most records (85.5%) originated from classes associated with warm to hot summers, reinforcing the well-known association between leptospirosis epidemiology and higher temperatures [33]. Reports from boreal and cold climates were uncommon; however, given the ongoing global climate shifts, leptospirosis should not be overlooked in these settings. Precipitation patterns were also noteworthy. More than half of the analyzed records (54.5%) came from areas classified as relatively dry, including savannah and steppe classes. The epidemiology of leptospirosis is often linked to humid, high-precipitation environments that favor environmental survival and transmission [34]. Nonetheless, bovine leptospirosis can also be transmitted through direct bovine-to-bovine route, particularly exposure to urine in high-density housing systems and venereal transmission [9,13]. It reduces the relative influence of humidity-precipitation and, to some extent, temperature. Overall, these findings support that bovine leptospirosis cannot be restricted to a single climate group or weather profile.

Regarding the genotyping methods herein used, it is noteworthy that most sequences originated from clinical samples, reflecting the widespread use of direct genotyping. In this context, single-locus sequencing enables the genotyping of clinical samples without prior culture by sequencing a genetic marker with sufficient taxonomic resolution. Although single-locus sequencing has been widely applied to animal leptospirosis and can support epidemiological inference and surveillance, the lack of standardized target markers remains a key limitation. In our dataset, eight markers were used for sequencing, either alone or in combination: *secY* (preprotein translocase), *rrs* (16S rRNA), *lfb1* (fibronectin-binding protein), *rpoB* (RNA polymerase β subunit), *flaB* (flagellar filament core protein), *lipL32* (outer membrane lipoprotein), *glmU* (bifunctional GlmU enzyme), and *gyrB* (DNA gyrase subunit B). The partial gene *secY* was the most frequently used marker and has been described as providing good taxonomic resolution [20]. The use of *rrs* (16S rRNA) was also common, as expected given its broad adoption in bacterial taxonomy and microbiome studies [35]. However, the use of multiple markers creates heterogeneous sequence databases, limiting cross-study comparisons because sequences can be compared only when generated from the same locus, a barrier to genetic surveillance. More studies are needed to systematically evaluate the taxonomic resolution of each marker and to define the most suitable marker for surveillance. Although this approach allows culture-independent genotyping, it is less reliable than whole-genome sequencing for taxonomic and epidemiological inference. Thus, results derived from single-locus sequencing should be interpreted with caution, especially considering the marker selected and its taxonomic resolution.

Leptospiral isolation followed by more robust genetic approaches, such as multilocus sequence typing (MLST) and whole-genome sequencing (WGS), together with serological classification as a phenotypic approach, remains the ideal strategy for comprehensive taxonomic identification. Beyond taxonomy, these methods also support inferences on the circulating clonal complexes and provide additional information, including the presence of virulence-associated factors and antimicrobial resistance genes [36,37,38]. However, given the intrinsic difficulty of culturing leptospires, particularly from animal samples, isolates were less frequent in our dataset than records obtained through direct genotyping. In our dataset, only 63 leptospiral genomes from bovines were deposited, whereas 255 isolates were described and genetically characterized using single-locus sequencing and/or MLST. This pattern once more highlights both the limited availability of isolates and the costs associated with whole-genome sequencing, particularly in low- and middle-income countries, where bovine leptospirosis is most frequently investigated.

Serological classification provides phenotypic characterization by enabling the identification of serogroups and serovars that cannot be determined by direct genotyping alone. In this study, the isolates were classified into 14 serogroups, with Sejroe (40.7%) as the predominant one, as expected, as this serogroup is host-adapted to bovines and is known as a leading cause of reproductive failures, especially subclinical ones, in this species [39]. Nevertheless, when incidental serogroups were grouped as “non-Sejroe”, their overall frequency was higher than expected from an epidemiological perspective, even in tropical settings, where greater serogroup diversity may be more easily explained. This pattern may be partially attributed to the fastidious growth and challenging isolation of Sejroe strains, particularly Hardjo genotypes. In addition, transient urinary shedding of different incidental leptospires has been reported in previous studies conducted in tropical areas, raising questions about the broader adaptability of leptospires to cattle [40], as well as the possible occurrence of coinfections involving different serovars. The high proportion of isolates from urine and kidney deposited in public databases may also have influenced this result.

Among the most common non-Sejroe serogroups identified herein, Pomona is known to be adapted to swine, whereas Icterohaemorrhagiae is adapted to rodents [41]. Grippotyphosa, on the other hand, has been isolated from more than 39 different animal species, suggesting the absence of a strict host [6]. Although transient and possibly chronic shedding of incidental strains by cattle remains poorly characterized, this is a highly relevant topic because vaccine formulations are based on serogroup representation and protective immunity is largely serogroup-specific [16]. Understanding the serogroup framework in a specific area not only enables epidemiological inferences but also improves vaccine refinement. Therefore, these findings indicate that vaccine development and refinement should be guided by periodic epidemiological surveillance of circulating leptospiral serovars, particularly in areas where non-Sejroe serogroups are frequently detected.

Finally, regarding host aspects, there was a notable difference in the origin of samples used for genotyping or isolation. The majority of leptospiral records were from urine or kidney tissues from bovines (~70%), highlighting the historical emphasis on the renal tract as the main target of leptospiral tropism. Genital colonization had already been demonstrated in the 1980s [14,42] and is a key aspect of Bovine Genital Leptospirosis, which was formally described only in 2020 [15]. Since then, genital samples such as cervicovaginal mucus, uterine fragments, and semen have been increasingly used for diagnosis and genotyping [13,43]. However, few isolates have been obtained from these sites and, consequently, few genomes have been generated to provide a better understanding of the differences between the two infections sites in cattle (renal vs. genital) and to confirm site preferences of specific strains, such as the reported preference of Sejroe for uterine colonization. Therefore, more studies should prioritize genital sampling for isolation and subsequent genomic analyses. The clinical status of the animals studied was the least available information in the metadata. Most studies were conducted in slaughtered animals, which did not present systemic manifestations such as jaundice and hemorrhages. However, these are uncommon signs in adult cattle, and most studies did not retrieve the reproductive history of the slaughtered animals. Bovine leptospirosis, especially BGL, may remain clinically silent, and it is better interpreted when genital detection is associated with herd reproductive records and clinical data [9]. In this context, it is important to link reproductive history or other clinical information to genotyping results to assess whether a given haplotype or genotype is associated with similar outcomes across different locations. Therefore, field studies with detailed clinical and reproductive data remain essential and must be encouraged.

This study has some limitations that should be considered when interpreting the findings. First, the analysis was based on publicly available sequence and genome records, which are opportunistic, heterogeneous, and unevenly distributed across countries and regions. Therefore, the observed patterns should not be interpreted as direct estimates of prevalence, geographic burden, or population-level serogroup distribution. Second, many records corresponded to single-locus sequences, for which standardized database-level quality assessment and metadata completeness are often limited when compared with whole-genome records. Third, the available metadata were frequently incomplete, particularly regarding clinical presentation, sample source, host characteristics, herd-level information, vaccination status, and environmental conditions. As a result, we could not fully assess how host-related, management, ecological, or climatic factors influence leptospiral detection and transmission in cattle. Finally, the limited number of publicly available bovine isolates and genomes, especially from genital and other underrepresented anatomical sites, restricts broader conclusions about tissue tropism, serogroup diversity, and implications for surveillance or vaccine refinement. Thus, the patterns reported here should be interpreted more as hypothesis-generating and as a baseline to guide future standardized, geographically representative genomic and epidemiological studies.

## 5. Conclusions

This available records indicate a worldwide distribution of bovine leptospiral infection, with the predominance of *L. interrogans* and *L. borgpetersenii*, as expected, but also a broader heterogeneity among other species and circulating serogroups. Climate analysis demonstrated high diversity across tropical and temperate regions and supported leptospiral transmission among cattle in dry and low-humidity climates. This study also highlighted a concerning lack of standardization of genetic markers for single-locus sequencing and the low availability of isolates and genomes. In addition, it reflected a predominance of renal instead of genital samples and scarce reporting of clinical and reproductive information. Future approaches should include strengthening culture efforts (especially from genital samples), expanding whole-genome sequencing, and improving metadata regarding clinical signs. These strategies will be essential to support evidence-based vaccine evaluation and provide a better understanding of the role of specific haplotypes/genotypes in bovine leptospirosis.

## Figures and Tables

**Table 1:** Genotyping methods and genetic markers used for characterization of *Leptospira* spp. from bovine samples.

**Genotyping Method**			
**Identification**	**Clinical Sample**	**Cultured Isolates**	**Total of Records**
Single-locus sequencing (SLS)	306	105	411
WGS	0	63	63
cgMLST + Single-locus sequencing *	0	46	46
MLST + Single-locus sequencing *	6	27	33
MLST	0	16	16
**Total**	**314**	**255**	**569**
**Genetic Markers**			
**Target**	**Clinical Sample**	**Cultured Isolates**	**Total of records**
*secY*	171	163	334
16S rRNA (*rrs*)	31	73	104
*lfb1*	45	45	90
*rpoB*	33	0	33
*flaB*	20	0	20
*lipL32*	9	10	19
*gmlU*	10	0	10
*gyrB*	6	1	7

* Both methods were used simultaneously to genetically characterize the same sample or isolate.

**Table 2:** Distribution of the main leptospiral species (N > 20 records) according to the identification methodology and corresponding methodological weight.

Main Species	N	Mean Weight	Distribution of Records		
			WGS or cgMLST	MLST	SLS
*L. borgpetersenii*	194	1.38	32 (29.6%)	10 (20.4%)	152 (36.9%)
*L. interrogans*	208	1.47	42 (38.9%)	13 (26.5%)	153 (37.1%)
*L. kirschneri*	36	1.28	5 (4.6%)	0	31 (7.5%)
*L. noguchii*	28	02.04	10 (9.3%)	9 (18.4%)	9 (2.2%)
*L. santarosai*	49	1.92	14 (13%)	17 (34.7%)	18 (4.4%)

WGS, whole-genome sequencing; cgMLST, core-genome multilocus sequence typing; MLST, multilocus sequence typing; SLS, single-locus sequence analysis. Methodological weights were assigned according to the expected taxonomic resolution of each approach: WGS/cgMLST = 3, MLST = 2, and SLS = 1. Percentages indicate the proportion of each species within each methodological category.

**Table 3:** Distribution of records of *Leptospira* spp. from bovines by geographic region and climate using Köppen–Geiger classification.

Continent	Geographic Subregion	N of Records by Region	Total Records by Continent
Africa	Eastern Africa	70	76
	Western Africa	6	
Americas	Caribbean	8	362
	Central America	3	
	Northern America	23	
	South America	328	
Asia	Central Asia	2	76
	Eastern Asia	2	
	Western Asia	8	
	Southeast Asia	12	
	Southern Asia	52	
Europe	Northern Europe	6	36
	Southern Europe	18	
	Western Europe	12	
**Main climate**	**Köppen–Geiger (KG) classes**	**N of records by KG class**	**Total of records by main climate**
Tropical	Aw—Tropical dry winter (tropical savannah climate)	170	303
	Am—Tropical monsoon climate	86	
	Af—Tropical fully humid (tropical rainforest climate)	47	
Temperate	Csc—Temperate dry winter short summer	57	135
	Cfa—Temperate fully humid hot summer (humid subtropical climate)	47	
	Cfb—Temperate fully humid warm summer (oceanic climate)	16	
	Cwa—Temperate dry winter, hot summer (humid subtropical climate)	14	
	Cwb—Temperate dry winter warm summer (subtropical highland climate)	1	
Steppe	BSh—Hot semi-arid (steppe climate)	3	3
Mediterranean	Csa—Hot-summer Mediterranean climate	15	15
Continental-Cold	Dfa—Continental/cold fully humid hot summer	12	12
Climate not defined (insufficient data)		101	

## Data Availability

The raw data supporting the conclusions of this article will be made available by the authors on request.

## Supplementary Materials

The following supporting information can be downloaded at: https://www.mdpi.com/article/10.3390/ani16132017/s1. Table S1. Full metadata of bovine *Leptospira* sequences and genomes deposited in GenBank and BIGSdb-Pasteur; Table S2. Distribution of bovine *Leptospira* records by country; Table S3. Bovine-origin *Leptospira* genome and sequence records stratified into South America and other countries/continents to minimize potential misclassification biases; Table S4. Temporal distribution of sequence and genome records of *Leptospira* spp. from bovine deposited in public datasets.

## Abbreviations

The following abbreviations are used in this manuscript:

BGL	Bovine Genital Leptospirosis
MAT	Microscopic Agglutination Test
MLST	Multi-Locus Sequence Typing
SLS	Single-locus Sequencing
WGS	Whole Genome Sequencing
