# Supplementary material for: Genetic Epidemiology of Bovine Leptospirosis: A Global Perspective from Sequence and Genome Datasets
Source: Animals (Basel). 2026 Jul 2;16(13):2017. doi: 10.3390/ani16132017 (PMC13359918; doi:10.3390/ani16132017)
Supplement: Supplementary file 1 [file animals-16-02017-s001.zip › Supplementary Table S1.pdf]

Table S1 - Full metadata of bovine *Leptospira* sequences and genomes deposited in GenBank and BIGSdb-Pasteur

GB accession	*Leptospira* Species	Strain/Sample ID	*Gene*	Serovar/Serogroup	Material Type	Origin sample	Clinical signs	Geographical location (state and country)	Continent	Geographic Regions	Climate (Köppen-Geiger)	Reference
AB563500.1	*L. interrogans*	SLCU49	*flaB*	-	Clinical sample	Urine	ND - Slaughterhouse	Colombo, Sri Lanka	Asia	Southern Asia	Af	Gamage et al., 2014
AB699318.1	*L. interrogans*	SLCU-10C5	*flaB*	-	Clinical sample	Urine	ND - Slaughterhouse	Colombo, Sri Lanka	Asia	Southern Asia	Af	Gamage et al., 2014
AB699319.1	*Leptospira* sp.	SLCU-10C15	*flaB*	-	Clinical sample	Urine	ND - Slaughterhouse	Colombo, Sri Lanka	Asia	Southern Asia	Af	Gamage et al., 2014
AB699320.1	*Leptospira sp.*	SLCU-10C20	*flaB*	-	Clinical sample	Urine	ND - Slaughterhouse	Colombo, Sri Lanka	Asia	Southern Asia	Af	Gamage et al., 2014
AB699321.1	*Leptospira sp.*	SLCU-10C24	*flaB*	-	Clinical sample	Urine	ND - Slaughterhouse	Colombo, Sri Lanka	Asia	Southern Asia	Af	Gamage et al., 2014
AB699322.1	*L. interrogans*	SLCU-10C36	*flaB*	-	Clinical sample	Urine	ND - Slaughterhouse	Colombo, Sri Lanka	Asia	Southern Asia	Af	Gamage et al., 2014
AB699323.1	*L. borgpetersenii*	SLCU-10C38	*flaB*	-	Clinical sample	Urine	ND - Slaughterhouse	Colombo, Sri Lanka	Asia	Southern Asia	Af	Gamage et al., 2014
AB699324.1	*L. interrogans*	SLCU-10C44	*flaB*	-	Clinical sample	Urine	ND - Slaughterhouse	Colombo, Sri Lanka	Asia	Southern Asia	Af	Gamage et al., 2014
AB699325.1	*L. borgpetersenii*	SLCU-10C52	*flaB*	-	Clinical sample	Urine	ND - Slaughterhouse	Colombo, Sri Lanka	Asia	Southern Asia	Af	Gamage et al., 2014

Table S1 - Full metadata of bovine *Leptospira* sequences and genomes deposited in GenBank and BIGSdb-Pasteur

AB699326.1	*Leptospira sp.*	SLCU-10C53	*flaB*	-	Clinical sample	Urine	ND - Slaughterhouse	Colombo, Sri Lanka	Asia	Southern Asia	Af	Gamage et al., 2014
AB699327.1	*L. borgpetersenii*	SLCU-10C54	*flaB*	-	Clinical sample	Urine	ND - Slaughterhouse	Colombo, Sri Lanka	Asia	Southern Asia	Af	Gamage et al., 2014
AB699329.1	*L. borgpetersenii*	SLCU-10C72	*flaB*	-	Clinical sample	Urine	ND - Slaughterhouse	Colombo, Sri Lanka	Asia	Southern Asia	Af	Gamage et al., 2014
AB699330.1	*L. borgpetersenii*	SLCU-10C74	*flaB*	-	Clinical sample	Urine	ND - Slaughterhouse	Colombo, Sri Lanka	Asia	Southern Asia	Af	Gamage et al., 2014
AB699331.1	*Leptospira sp.*	SLCU-10C75	*flaB*	-	Clinical sample	Urine	ND - Slaughterhouse	Colombo, Sri Lanka	Asia	Southern Asia	Af	Gamage et al., 2014
AB699332.1	*L. borgpetersenii*	SLCU-10C80	*flaB*	-	Clinical sample	Urine	ND - Slaughterhouse	Colombo, Sri Lanka	Asia	Southern Asia	Af	Gamage et al., 2014
AB699333.1	*L. borgpetersenii*	SLCU-10C110	*flaB*	-	Clinical sample	Urine	ND - Slaughterhouse	Colombo, Sri Lanka	Asia	Southern Asia	Af	Gamage et al., 2014
AB699334.1	*L. borgpetersenii*	SLCU-10C113	*flaB*	-	Clinical sample	Urine	ND - Slaughterhouse	Colombo, Sri Lanka	Asia	Southern Asia	Af	Gamage et al., 2014
AB699335.1	*L. borgpetersenii*	SLCU-10C135	*flaB*	-	Clinical sample	Urine	ND - Slaughterhouse	Colombo, Sri Lanka	Asia	Southern Asia	Af	Gamage et al., 2014
AB699336.1	*Leptospira* sp.	SLCU-10C138	*flaB*	-	Clinical sample	Urine	ND - Slaughterhouse	Colombo, Sri Lanka	Asia	Southern Asia	Af	Gamage et al., 2014
AB699337.1	*Leptospira* sp.	SLCU-10C144	*flaB*	-	Clinical sample	Urine	ND - Slaughterhouse	Colombo, Sri Lanka	Asia	Southern Asia	Af	Gamage et al., 2014

Table S1 - Full metadata of bovine *Leptospira* sequences and genomes deposited in GenBank and BIGSdb-Pasteur

DQ286415.1	*L. borgpetersenii*	MGA09	*lipL32*	-	Clinical sample	Urine	Reproductive failures	Minas Gerais, Brazil	America	South America	Aw	Bomfim and Koury, 2006
DQ286416.1	*L. borgpetersenii*	MGA28	*lipL32*	-	Clinical sample	Urine	Reproductive failures	Minas Gerais, Brazil	America	South America	Aw	Bomfim and Koury, 2006
DQ286417.1	*L. borgpetersenii*	MGA52	*lipL32*	-	Clinical sample	Urine	Reproductive failures	Minas Gerais, Brazil	America	South America	Aw	Bomfim and Koury, 2006
DQ286418.1	*L. interrogans*	MGA182	*lipL32*	-	Clinical sample	Urine	Reproductive failures	Minas Gerais, Brazil	America	South America	Aw	Bomfim and Koury, 2006
DQ343231.1	*L. borgpetersenii*	Lely607	*lipL32*	Hardjo/Sejroe	Isolate	Urine	Reproductive failures	Minas Gerais, Brazil	America	South America	Aw	Bomfim and Koury, 2006
DQ483058.1	*L. weilli*	94-79970/3	16S rRNA + *gyrB*	Topaz/Tarassovi	Isolate	Urine	ND	Queensland, Australia	Oceania	Australia and New Zealand	BSh	Corney et al., 2008
FJ434138.1	*Leptospira* sp.	BDUCU6	*secY*	-	Clinical sample	Urine	ND	ND, India	Asia	Southern Asia	ND	Unpublished
GCA_001276935.1	*L. noguchii*	U73/2013	WGS	ND/Panama	Isolate	Urine	ND - Slaughterhouse	Rio de Janeiro, Brazil	America	South America	Aw	Martins et al., 2015
GCA_001952675.1	*L. santarosai*	LO-9	cgMLST + *lfb1* + *secY*	Grippotyphosa/Grippotyphosa	Isolate	Urine	ND	ND, Brazil	America	South America	ND	Unpublished
GCA_023515975.1	*L. interrogans*	N116	WGS	Hardjo/Sejroe	Isolate	Vaginal mucus	ND	ND, Belgium	Europe	Western Europe	Cfa	Unpublished
GCA_025792475.2	*L. borgpetersenii*	Vache K3	WGS	Hardjo/Sejroe	Isolate	Kidney	ND	ND, Belgium	Europe	Western Europe	Cfa	Unpublished
GCA_048974055.1	*L. borgpetersenii*	BS-39	WGS	ND	Isolate	Semen	ND	Iowa, United States	America	Northern America	Dfa	Unpublished
GCF_000988105.2	*L. santarosai*	U160	WGS	ND/Sarmin	Isolate	Urine	ND - Slaughterhouse	Rio de Janeiro, Brazil	America	South America	Aw	Kremer et al., 2015
GCF_001008325.2	*L. santarosai*	U233	WGS	ND/Grippotyphosa	Isolate	Urine	ND - Slaughterhouse	Rio de Janeiro, Brazil	America	South America	Aw	Kremer et al., 2015

Table S1 - Full metadata of bovine *Leptospira* sequences and genomes deposited in GenBank and BIGSdb-Pasteur

GCF_001008335.2	*L. santarosai*	U164	WGS	ND/Tarassovi	Isolate	Urine	ND - Slaughterhouse	Rio de Janeiro, Brazil	America	South America	Aw	Kremer et al., 2015
GCF_001022315.1	*L. interrogans*	acegua	WGS	Muenchen/Australis	Isolate	Kidney (fetus)	ND - Slaughterhouse	Rio Grande do Sul, Brazil	America	South America	Csc	Monte et al., 2015
GCF_001293065.1	*L. interrogans*	Norma	WGS	Hardjo/Sejroe	Isolate	Urine	Reproductive failures	Minas Gerais, Brazil	America	South America	Aw	Cosate et al., 2015
GCF_001584245.1	*L. kirschneri*	ZV013	WGS	ND	Isolate	Urine	ND	Manabí, Ecuador	America	South America	BSh	Barragan et al., 2016
GCF_001584255.1	*L. interrogans*	ZV016	WGS	ND	Isolate	Urine	ND	Manabí, Ecuador	America	South America	BSh	Barragan et al., 2016
GCF_001618445.1	*L. borgpetersenii*	BK-30	WGS	Hardjo/Sejroe	Isolate	Kidney	ND - Slaughterhouse	Georgia, United States	America	Northern America	Csc	Llanes et al., 2018
GCF_001618485.1	*L. borgpetersenii*	NVSL S 818	WGS	Hardjo/Sejroe	Isolate	Kidney	ND	Texas, United States	America	Northern America	Csc	Llanes et al., 2018
GCF_001618525.1	*L. borgpetersenii*	BK-6	WGS	Hardjo/Sejroe	Isolate	Kidney	ND - Slaughterhouse	Georgia, United States	America	Northern America	Csc	Llanes et al., 2018
GCF_001618565.1	*L. borgpetersenii*	BK-9	WGS	Hardjo/Sejroe	Isolate	Kidney	ND - Slaughterhouse	Georgia, United States	America	Northern America	Csc	Llanes et al., 2018
GCF_001618585.1	*L. borgpetersenii*	NVSL S 1343	WGS	Hardjo/Sejroe	Isolate	Kidney	ND	Texas, United States	America	Northern America	Csc	Llanes et al., 2018
GCF_001857845.1	*L. interrogans*	AKRFB	WGS	ND/Pomona	Isolate	Kidney (fetus)	Abortion	Buenos Aires, Argentina	America	South America	Cfb	Varni et al., 2016
GCF_001952675.1	*L. santarosai*	LO-9	WGS	ND/Grippotyphosa	Isolate	Urine	ND - Slaughterhouse	Paraná, Brazil	America	South America	Csc	Unpublished
GCF_001952685.1	*L. santarosai*	M498	cgMLST + *lfb1* + *secY*	Guaricura/Sejroe	Isolate	Urine	ND	ND, Brazil	America	South America	ND	Unpublished
GCF_002009805.1	*L. alexanderi*	56643	WGS	Weaveri/Sarmin	Isolate	ND	ND	Yunnan, China	Asia	Eastern Asia	Cwb	Xu et al., 2016

Table S1 - Full metadata of bovine *Leptospira* sequences and genomes deposited in GenBank and BIGSdb-Pasteur

GCF_002150045.1	*L. venezuelensis*	IVIC-Bov1	WGS	ND	Isolate	Urine	ND	ND, Venezuela	America	South America	ND	Puche et al., 2018
GCF_003254845.1	*L. borgpetersenii*	203	WGS	Hardjo/Sejroe	Isolate	Kidney	ND	ND, United States	America	Northern America	ND	Unpublished
GCF_006568845.1	*L. noguchii*	IP1605021	WGS	ND/Pyrogenes	Isolate	Urine	ND - Slaughterhouse	Salto, Uruguay	America	South America	Cfa	Zarantonelli et al., 2018
GCF_006568855.1	*L. borgpetersenii*	IP1512012	*secY*	Hardjo/Sejroe	Isolate	Urine	ND - Slaughterhouse	Salto, Uruguay	America	South America	Cfa	Zarantonelli et al., 2018
GCF_006568855.1	*L. borgpetersenii*	IP1512012	WGS	Hardjo/Sejroe	Isolate	Urine	ND - Slaughterhouse	Salto, Uruguay	America	South America	Cfa	Zarantonelli et al., 2018
GCF_006568865.1	*L. borgpetersenii*	IP1506001	WGS	Hardjo/Sejroe	Isolate	Urine	ND - Slaughterhouse	Canelones, Uruguay	America	South America	Csc	Zarantonelli et al., 2018
GCF_006568875.1	*L. interrogans*	IP1512015	WGS	Kennewicki/Pomona	Isolate	Urine	ND - Slaughterhouse	Artigas, Uruguay	America	South America	Csc	Zarantonelli et al., 2018
GCF_006568885.1	*L. interrogans*	IP1512016	WGS	Kennewicki/Pomona	Isolate	Urine	ND - Slaughterhouse	Artigas, Uruguay	America	South America	Csc	Zarantonelli et al., 2018
GCF_006568945.1	*L. interrogans*	IP1507003	WGS	Hardjo/Sejroe	Isolate	Urine	ND - Slaughterhouse	Paysandú, Uruguay	America	South America	Csc	Zarantonelli et al., 2018
GCF_006569045.1	*L. interrogans*	IP1512014	WGS	Kennewicki/Pomona	Isolate	Urine	ND - Slaughterhouse	Artigas, Uruguay	America	South America	Csc	Zarantonelli et al., 2018
GCF_008118365.1	*L. interrogans*	L53	WGS	Hardjo/Sejroe	Isolate	Urine	Reproductive failures	Paraná, Brazil	America	South America	Csc	Chideroli et al., 2017
GCF_017653725.1	*L. interrogans*	R287	WGS	Copenhageni/Icterohaemorrhagiae	Isolate	Fetus	Abortion	ND, Ireland	Europe	Northern Europe	Cfb	Unpublished

Table S1 - Full metadata of bovine *Leptospira* sequences and genomes deposited in GenBank and BIGSdb-Pasteur

GCF_017653855.1	*L. interrogans*	S606	WGS	Copenhageni/Icterohaemorrhagiae	Isolate	Lung	ND	ND, United Kingdom	Europe	Northern Europe	Cfb	Unpublished
GCF_019173505.1	*L. interrogans*	s147	WGS	ND	Isolate	ND	ND	Sardinia, Italy	Europe	Southern Europe	Csa	Unpublished
GCF_021014375.1	*L. interrogans*	Che_2014	WGS	ND	Isolate	Urine	ND	Tamil Nadu, India	Asia	Southern Asia	Aw	Unpublished
GCF_022436585.1	*L. borgpetersenii*	KR83	WGS	Hardjo/Sejroe	Isolate	Urine	ND	ND, United Kingdom	Europe	Northern Europe	Cfb	Unpublished
GCF_022436615.1	*L. borgpetersenii*	KR39	WGS	Hardjo/Sejroe	Isolate	Urine	ND	ND, United Kingdom	Europe	Northern Europe	Cfb	Unpublished
GCF_022819405.1	*L. noguchii*	IP1712055	WGS	ND	Isolate	Urine	ND - Slaughterhouse	Paysandú, Uruguay	America	South America	Csc	Zarantonelli et al., 2018
GCF_022819425.1	*L. noguchii*	IP1709037	WGS	ND/Autumnalis	Isolate	Kidney	ND - Slaughterhouse	Cerro Largo, Uruguay	America	South America	Csc	Zarantonelli et al., 2018
GCF_022819445.1	*L. noguchii*	IP1705032	WGS	ND/Autumnalis	Isolate	Urine	ND - Slaughterhouse	Florida, Uruguay	America	South America	Csc	Zarantonelli et al., 2018
GCF_022819565.1	*L. noguchii*	IP1703027	WGS	ND	Isolate	Urine	ND - Slaughterhouse	Durazno, Uruguay	America	South America	Csc	Zarantonelli et al., 2018
GCF_022819715.1	*L. noguchii*	IP1611024	WGS	ND/Australis	Isolate	Urine	ND - Slaughterhouse	Artigas, Uruguay	America	South America	Csc	Zarantonelli et al., 2018
GCF_022820045.1	*L. noguchii*	IP1512017	WGS	ND	Isolate	Urine	ND - Slaughterhouse	Florida, Uruguay	America	South America	Csc	Zarantonelli et al., 2018
GCF_022820565.1	*L. noguchii*	IP1804061	WGS	ND	Isolate	Urine	ND - Slaughterhouse	ND, Uruguay	America	South America	Csc	Nieves et al., 2022
GCF_023023725.1	*L. borgpetersenii*	TC112	WGS	Hardjo/Sejroe	Isolate	Urine	ND - Slaughterhouse	Iowa, United States	America	Northern America	Dfa	Putz et al., 2022

Table S1 - Full metadata of bovine *Leptospira* sequences and genomes deposited in GenBank and BIGSdb-Pasteur

GCF_023023745.1	*L. borgpetersenii*	TC129	WGS	Hardjo/Sejroe	Isolate	Urine	ND	Iowa, United States	America	Northern America	Dfa	Putz et al., 2022
GCF_023023865.1	*L. borgpetersenii*	TC147	WGS	Hardjo/Sejroe	Isolate	Urine	ND - Slaughterhouse	Iowa, United States	America	Northern America	Dfa	Putz et al., 2022
GCF_023023985.1	*L. borgpetersenii*	TC273	WGS	Hardjo/Sejroe	Isolate	Urine	ND - Slaughterhouse	Iowa, United States	America	Northern America	Dfa	Putz et al., 2022
GCF_024704545.1	*L. borgpetersenii*	MN900	WGS	ND/Tarassovi	Isolate	Urine	ND	Iowa, United States	America	Northern America	Dfa	Unpublished
GCF_026914205.1	*L. borgpetersenii*	DCP-009	WGS	ND	Isolate	Urine	ND	ND, Puerto Rico	America	Caribbean	Af	Unpublished
GCF_026914225.1	*L. borgpetersenii*	DCP-041	WGS	ND	Isolate	Urine	ND	ND, Puerto Rico	America	Caribbean	Af	Unpublished
GCF_026914325.1	*L. santarosai*	DCP-017	WGS	ND	Isolate	Urine	ND	ND, Puerto Rico	America	Caribbean	Af	Unpublished
GCF_030546555.1	*L. santarosai*	VF237	WGS	Guaricura/Sejroe	Isolate	Vaginal mucus	ND - Slaughterhouse	Rio de Janeiro, Brazil	America	South America	Aw	Di Azevedo et al., 2023
GCF_030546585.1	*L. santarosai*	2013_VF52	WGS	Guaricura/Sejroe	Isolate	Vaginal mucus	ND - Slaughterhouse	Rio de Janeiro, Brazil	America	South America	Aw	Di Azevedo et al., 2023
GCF_030931075.1	*L. interrogans*	22	WGS	ND	Isolate	Urine	ND	Almaty, Kazakhstan	Asia	Central Asia	Dfa	Unpublished
GCF_037000625.1	*L. venezuelensis*	IVIC-Bov3	WGS	ND	Isolate	ND	ND	ND, Venezuela	America	South America	ND	Unpublished
GCF_037000705.1	*L. venezuelensis*	IVIC.BOV 4	WGS	ND	Isolate	ND	ND	ND, Venezuela	America	South America	ND	Unpublished
GCF_042138915.1	*L. wolffii*	Lep080	WGS	ND	Isolate	Kidney	ND	Nariño, Colombia	America	South America	Cfb	Unpublished
GCF_042138935.1	*L. interrogans*	Lep037	WGS	-	Clinical sample	Fetus	ND	Cundinamarca, Colômbia	America	South America	Cfb	Unpublished
GCF_042138955.1	*L. interrogans*	ZV016	WGS	-	Clinical sample	Kidney	ND	Cundinamarca, Colômbia	America	South America	Cfb	Unpublished

Table S1 - Full metadata of bovine *Leptospira* sequences and genomes deposited in GenBank and BIGSdb-Pasteur

GCF_048973995.1	*L. borgpetersenii*	BU-20	WGS	ND	Isolate	Urine	ND	Iowa, United States	America	Northern America	Dfa	Unpublished
GCF_048974015.1	*L. borgpetersenii*	BU-18	WGS	ND	Isolate	Urine	ND	Iowa, United States	America	Northern America	Dfa	Unpublished
GCF_048974035.1	*L. borgpetersenii*	BU-19	WGS	ND	Isolate	Urine	ND	Iowa, United States	America	Northern America	Dfa	Unpublished
GCF_052161685.1	*L. interrogans*	L7	WGS	ND/Icterohaemorrhagiae	Isolate	ND	ND	Almaty, Kazakhstan	Asia	Central Asia	Dfa	Unpublished
HM046993.1	*L. interrogans*	ADMAS 1345	*rpoB*	-	Clinical sample	Blood	Systemic (Pyrexia)	ND, India	Asia	Southern Asia	ND	Balamurugan et al., 2013
HM046994.1	*L. interrogans*	ADMAS 1590	*rpoB*	-	Clinical sample	Serum	Reproductive failures	ND, India	Asia	Southern Asia	ND	Balamurugan et al., 2013
HM046996.1	*L. kirschneri*	ADMAS G73	*rpoB*	-	Clinical sample	Serum	Systemic (Pyrexia)	ND, India	Asia	Southern Asia	ND	Balamurugan et al., 2013
ID 1116	*L. kirschneri*	202001908	cgMLST + 16s rRNA + *lfb1* + *secY*	Sokoine/Icterohaemorrhagiae	Isolate	ND	ND	ND, Tanzania	Africa	Eastern Africa	ND	Unpublished
ID 1141	*L. interrogans*	202100512	cgMLST + 16s rRNA + *lfb1* + *secY*	ND	Isolate	ND	ND	ND, France	Europe	Western Europe	Cfa	Unpublished
ID 1438	*L. interrogans*	MH4000	cgMLST + *lfb1* + *secY*	ND/Pomona	Isolate	Urine	Reproductive failures	ND, Israel	Asia	Western Asia	Csa	Unpublished
ID 1439	*L. interrogans*	MH4001	cgMLST + 16s rRNA + *lfb1* + *secY*	ND/Canicola	Isolate	Urine	Reproductive failures	ND, Israel	Asia	Western Asia	Csa	Unpublished
ID 1440	*L. interrogans*	MH4006	cgMLST + *lfb1* + *secY*	ND	Isolate	Urine	Reproductive failures	ND, Israel	Asia	Western Asia	Csa	Unpublished
ID 1441	*L. interrogans*	MH4012	cgMLST + *lfb1* + *secY*	ND/Pomona	Isolate	Urine	Reproductive failures	ND, Israel	Asia	Western Asia	Csa	Unpublished
ID 1442	*L. interrogans*	MH4016	cgMLST + *lfb1* + *secY*	ND	Isolate	Urine	Reproductive failures	ND, Israel	Asia	Western Asia	Csa	Unpublished
ID 1443	*L. interrogans*	MH4027	cgMLST + 16s rRNA + *lfb1* + *secY*	ND/Icterohaemorrhagiae	Isolate	Urine	Reproductive failures	ND, Israel	Asia	Western Asia	Csa	Unpublished

Table S1 - Full metadata of bovine *Leptospira* sequences and genomes deposited in GenBank and BIGSdb-Pasteur

ID 1444	*L. interrogans*	MH4029	cgMLST + *lfb1* + *secY*	ND/Pomona	Isolate	Urine	Reproductive failures	ND, Israel	Asia	Western Asia	Csa	Unpublished
ID 1598	*L. borgpetersenii*	70_IZSLER	cgMLST + 16s rRNA + *lfb1* + *secY*	ND	Isolate	Urine	ND	ND, Italy	Europe	Southern Europe	Csa	Unpublished
ID 1599	*L. borgpetersenii*	112_IZSLER	cgMLST + 16s rRNA + *lfb1* + *secY*	Hardjo/Sejroe	Isolate	Kidney	ND	ND, Italy	Europe	Southern Europe	Csa	Unpublished
ID 792	*L. interrogans*	leptoCuba21/LCF453	cgMLST + 16s rRNA + *lfb1* + *secY*	Canicola/Canicola	Isolate	Urine	ND	ND, Cuba	America	Caribbean	Aw	Unpublished
ID 794	*L. interrogans*	leptoCuba23/LCF2004	cgMLST + 16s rRNA + *lfb1* + *secY*	Canicola/Canicola	Isolate	Urine	ND	ND, Cuba	America	Caribbean	Aw	Unpublished
ID 795	*L. interrogans*	leptoCuba24/LCF332	cgMLST + 16s rRNA + *lfb1* + *secY*	Canicola/Canicola	Isolate	Urine	ND	ND, Cuba	America	Caribbean	Aw	Unpublished
ID 922	*L. interrogans*	HO0905CK	cgMLST + 16s rRNA + *lfb1* + *secY*	Pomona/Pomona	Isolate	Kidney	ND	New Zealand	Oceania	Australia and New Zealand	Cfa	Unpublished
ID 923	*L. borgpetersenii*	HO0907CK	cgMLST + 16s rRNA + *lfb1* + *secY*	Hardjo/Sejroe	Isolate	Kidney	ND	New Zealand	Oceania	Australia and New Zealand	Cfa	Unpublished
ID 924	*L. borgpetersenii*	HO0912CK	cgMLST + 16s rRNA + *lfb1* + *secY*	Hardjo/Sejroe	Isolate	Kidney	ND	New Zealand	Oceania	Australia and New Zealand	Cfa	Unpublished
JF718726.1	*Leptospira* sp.	ADMAS272	*rpoB*	-	Clinical sample	Serum	ND	Maharashtra, India	Asia	Southern Asia	Aw	Unpublished
JF718728.1	*Leptospira* sp.	ADMAS278	*rpoB*	-	Clinical sample	Serum	ND	Maharashtra, India	Asia	Southern Asia	Aw	Unpublished
JF718731.1	*Leptospira* sp.	ADMAS267	*rpoB*	-	Clinical sample	Serum	ND	Maharashtra, India	Asia	Southern Asia	Aw	Unpublished
JF718738.1	*Leptospira* sp.	ADMAS338	*rpoB*	-	Clinical sample	Vaginal mucus	Reproductive failures	Karnataka, India	Asia	Southern Asia	Aw	Balamurugan et al., 2013

Table S1 - Full metadata of bovine *Leptospira* sequences and genomes deposited in GenBank and BIGSdb-Pasteur

JF718739.1	*Leptospira* sp.	ADMAS340	*rpoB*	-	Clinical sample	Vaginal mucus	Reproductive failures	Karnataka, India	Asia	Southern Asia	Aw	Balamurugan et al., 2013
JN388617.1	*L. interrogans*	G70	*rpoB*	-	Clinical sample	Serum	Reproductive failures	ND, India	Asia	Southern Asia	ND	Balamurugan et al., 2013
JN388619.1	*L. interrogans*	ADMAS 1228	*rpoB*	-	Clinical sample	Serum	Systemic (Pyrexia)	ND, India	Asia	Southern Asia	ND	Balamurugan et al., 2013
JN388621.1	*Leptospira* sp.	ADMAS G202	*rpoB*	-	Clinical sample	Peritoneal fluid (fetus)	Abortion	ND, India	Asia	Southern Asia	ND	Balamurugan et al., 2013
JN388622.1	*Leptospira* sp.	ADMAS G229	*rpoB*	-	Clinical sample	Blood (fetus)	Abortion	ND, India	Asia	Southern Asia	ND	Balamurugan et al., 2013
JN388623.1	*L. interrogans*	G90	*rpoB*	-	Clinical sample	Serum	Reproductive failures	ND, India	Asia	Southern Asia	ND	Balamurugan et al., 2013
JN388631.1	*Leptospira* sp.	ADMAS 2421	*rpoB*	-	Clinical sample	Serum	ND	ND, India	Asia	Southern Asia	ND	Balamurugan et al., 2013
JN388632.1	*L. interrogans*	ADMAS 1856	*rpoB*	-	Clinical sample	Serum	ND	ND, India	Asia	Southern Asia	ND	Balamurugan et al., 2013
JN388635.1	*L. interrogans*	ADMAS 2475	*rpoB*	-	Clinical sample	Serum	ND	ND, India	Asia	Southern Asia	ND	Balamurugan et al., 2013
JN388636.1	*Leptospira* sp.	ADMAS 421	*rpoB*	-	Clinical sample	Serum	ND	ND, India	Asia	Southern Asia	ND	Balamurugan et al., 2013
JN388637.1	*Leptospira* sp.	ADMAS 3360	*rpoB*	-	Clinical sample	Blood	ND	ND, India	Asia	Southern Asia	ND	Balamurugan et al., 2013
JN388639.1	*Leptospira* sp.	ADMAS 2757	*rpoB*	-	Clinical sample	Serum	ND	ND, India	Asia	Southern Asia	ND	Balamurugan et al., 2013
JN388640.1	*Leptospira* sp.	ADMAS 3334	*rpoB*	-	Clinical sample	Serum	Systemic (Pyrexia)	ND, India	Asia	Southern Asia	ND	Balamurugan et al., 2013
JN388642.1	*Leptospira* sp.	ADMAS 2882	*rpoB*	-	Clinical sample	Serum	ND	ND, India	Asia	Southern Asia	ND	Balamurugan et al., 2013
JN388647.1	*Leptospira* sp.	ADMAS 2480	*rpoB*	-	Clinical sample	Serum	ND	ND, India	Asia	Southern Asia	ND	Balamurugan et al., 2013
JN388651.1	*Leptospira* sp.	ADMAS 2779	*rpoB*	-	Clinical sample	Serum	ND	ND, India	Asia	Southern Asia	ND	Balamurugan et al., 2013

Table S1 - Full metadata of bovine *Leptospira* sequences and genomes deposited in GenBank and BIGSdb-Pasteur

JN388651.1	*Leptospira* sp.	ADMAS 2779	*rpoB*	-	Clinical sample	Serum	ND	ND, India	Asia	Southern Asia	ND	Balamurugan et al., 2013
JN388653.1	*Leptospira* sp.	ADMAS 3377	*rpoB*	-	Clinical sample	Blood	ND	ND, India	Asia	Southern Asia	ND	Balamurugan et al., 2013
JQ765635.1	*L. borgpetersenii*	Mini-CTG	16S rRNA	-	Clinical sample	ND	ND	Minas Gerais, Brazil	America	South America	Aw	Unpublished
JQPC01000032.1	*L. interrogans*	20046	WGS	ND	Isolate	ND	ND	Sichuan, China	Asia	Eastern Asia	Cwa	Xu et al., 2016
KF184493.1	*L. interrogans*	Marcos Juarez	MLST scheme#2	ND/Pomona	Isolate	ND	ND	ND, Argentina	America	South America	ND	Varni et al., 2014
KF184494.1	*L. interrogans*	Fulton	MLST scheme#2	ND/Pomona	Isolate	ND	ND	ND, Argentina	America	South America	ND	Varni et al., 2014
KF184495.1	*L. interrogans*	Pujato	MLST scheme#2	ND/Pomona	Isolate	ND	ND	ND, Argentina	America	South America	ND	Varni et al., 2014
KF184503.1	*L. interrogans*	Corrientes 289	MLST scheme#2	ND/Pomona	Isolate	ND	ND	ND, Argentina	America	South America	ND	Varni et al., 2014
KF184504.1	*L. interrogans*	Rojas	MLST scheme#2	ND/Pomona	Isolate	ND	ND	ND, Argentina	America	South America	ND	Varni et al., 2014
KF184509.1	*L. interrogans*	Macedo Balcarce	MLST scheme#2	ND/Pomona	Isolate	ND	ND	ND, Argentina	America	South America	ND	Varni et al., 2014
KF184511.1	*L. interrogans*	Bayur P	MLST scheme#2	ND/Pomona	Isolate	ND	ND	ND, Argentina	America	South America	ND	Varni et al., 2014
KF184512.1	*L. interrogans*	Corrientes 266	MLST scheme#2	ND/Pomona	Isolate	ND	ND	ND, Argentina	America	South America	ND	Varni et al., 2014
KF184513.1	*L. interrogans*	Bayur C	MLST scheme#2	ND/Canicola	Isolate	ND	ND	ND, Argentina	America	South America	ND	Varni et al., 2014
KF184516.1	*L. interrogans*	Bobino bibi	MLST scheme#2	ND/Pomona	Isolate	ND	ND	ND, Argentina	America	South America	ND	Varni et al., 2014
KM042085.1	*L. borgpetersenii*	SHLEP	16S rRNA (*rrs*)	-	Clinical sample	Kidney	ND	ND, Iran	Asia	Southern Asia	ND	Unpublished
KP263062.1	*L. santarosai*	U152/2013	*secY*	-	Clinical sample	Urine	ND - Slaughterhouse	Rio de Janeiro, Brazil	America	South America	Aw	Pinto et al., 2015

Table S1 - Full metadata of bovine *Leptospira* sequences and genomes deposited in GenBank and BIGSdb-Pasteur

KP263066.1	*L. noguchii*	U232/2013	*secY*	ND/Autumnalis	Isolate	Urine	ND - Slaughterhouse	Rio de Janeiro, Brazil	America	South America	Aw	Martins et al., 2015
KP263068.1	*L. santarosai*	U278/2013	*secY* + MLST scheme #1	-	Clinical sample	Urine	ND - Slaughterhouse	Rio de Janeiro, Brazil	America	South America	Aw	Pinto et al., 2015
KP263069.1	*L. santarosai*	U280/2013	*secY* + MLST scheme #1	-	Clinical sample	Urine	ND - Slaughterhouse	Rio de Janeiro, Brazil	America	South America	Aw	Pinto et al., 2015
KP862625.1	*L. interrogans*	2013_U5	*secY*	-	Clinical sample	Urine	ND - Slaughterhouse	Rio de Janeiro, Brazil	America	South America	Aw	Hamond et al., 2015
KP862626.1	*L. interrogans*	2013_U16	*secY*	-	Clinical sample	Urine	ND - Slaughterhouse	Rio de Janeiro, Brazil	America	South America	Aw	Hamond et al., 2015
KP862628.1	*L. borgpetersenii*	2013_U83	*secY*	-	Clinical sample	Urine	ND - Slaughterhouse	Rio de Janeiro, Brazil	America	South America	Aw	Hamond et al., 2015
KP862629.1	*L. interrogans*	2013_U85	*secY*	-	Clinical sample	Urine	ND - Slaughterhouse	Rio de Janeiro, Brazil	America	South America	Aw	Hamond et al., 2015
KP862630.1	*L. borgpetersenii*	2013_U98	*secY*	-	Clinical sample	Urine	ND - Slaughterhouse	Rio de Janeiro, Brazil	America	South America	Aw	Hamond et al., 2015
KP862631.1	*L. borgpetersenii*	2013_U146	*secY*	-	Clinical sample	Urine	ND - Slaughterhouse	Rio de Janeiro, Brazil	America	South America	Aw	Hamond et al., 2015
KP862632.1	*L. santarosai*	2013_U152	*secY* + MLST scheme #1	-	Clinical sample	Urine	ND - Slaughterhouse	Rio de Janeiro, Brazil	America	South America	Aw	Hamond et al., 2015
KP862633.1	*L. santarosai*	2013_U160	*secY* + MLST scheme #1	-	Clinical sample	Urine	ND - Slaughterhouse	Rio de Janeiro, Brazil	America	South America	Aw	Hamond et al., 2015

Table S1 - Full metadata of bovine *Leptospira* sequences and genomes deposited in GenBank and BIGSdb-Pasteur

KP862634.1	*L. santarosai*	2013_U164	*secY* + MLST scheme #1	-	Clinical sample	Urine	ND - Slaughterhouse	Rio de Janeiro, Brazil	America	South America	Aw	Hamond et al., 2015
KP862635.1	*L. interrogans*	2013_U167	*secY*	-	Clinical sample	Urine	ND - Slaughterhouse	Rio de Janeiro, Brazil	America	South America	Aw	Hamond et al., 2015
KP862636.1	*L. borgpetersenii*	2013_U183	*secY*	-	Clinical sample	Urine	ND - Slaughterhouse	Rio de Janeiro, Brazil	America	South America	Aw	Hamond et al., 2015
KP862637.1	*L. interrogans*	2013_U193	*secY*	-	Clinical sample	Urine	ND - Slaughterhouse	Rio de Janeiro, Brazil	America	South America	Aw	Hamond et al., 2015
KP862638.1	*L. borgpetersenii*	2013_U194	*secY*	-	Clinical sample	Urine	ND - Slaughterhouse	Rio de Janeiro, Brazil	America	South America	Aw	Hamond et al., 2015
KP862639.1	*L. borgpetersenii*	2013_U215	*secY*	-	Clinical sample	Urine	ND - Slaughterhouse	Rio de Janeiro, Brazil	America	South America	Aw	Hamond et al., 2015
KP862640.1	*L. interrogans*	2013_U225	*secY*	-	Clinical sample	Urine	ND - Slaughterhouse	Rio de Janeiro, Brazil	America	South America	Aw	Hamond et al., 2015
KP862641.1	*L. noguchii*	2013_U232	*secY*	-	Clinical sample	Urine	ND - Slaughterhouse	Rio de Janeiro, Brazil	America	South America	Aw	Hamond et al., 2015
KP862642.1	*L. santarosai*	2013_U233	*secY* + MLST scheme #1	-	Clinical sample	Urine	ND - Slaughterhouse	Rio de Janeiro, Brazil	America	South America	Aw	Hamond et al., 2015
KP862643.1	*L. borgpetersenii*	2013_U275	*secY*	-	Clinical sample	Urine	ND - Slaughterhouse	Rio de Janeiro, Brazil	America	South America	Aw	Hamond et al., 2015
KP862644.1	*L. borgpetersenii*	2013_U277	*secY*	-	Clinical sample	Urine	ND - Slaughterhouse	Rio de Janeiro, Brazil	America	South America	Aw	Hamond et al., 2015

Table S1 - Full metadata of bovine *Leptospira* sequences and genomes deposited in GenBank and BIGSdb-Pasteur

KP862645.1	*L. santarosai*	2013_U278	*secY*	-	Clinical sample	Urine	ND - Slaughterhouse	Rio de Janeiro, Brazil	America	South America	Aw	Hamond et al., 2015
KP862646.1	*L. santarosai*	2013_U280	*secY*	-	Clinical sample	Urine	ND - Slaughterhouse	Rio de Janeiro, Brazil	America	South America	Aw	Hamond et al., 2015
KP862647.1	*L. borgpetersenii*	2013_U291	*secY*	-	Clinical sample	Urine	ND - Slaughterhouse	Rio de Janeiro, Brazil	America	South America	Aw	Hamond et al., 2015
KR030150.1	*L. interrogans*	Nigeria-PLA09/2013	16S rRNA	Copenhageni/Icterohaemorrhagiae	Isolate	Urine	ND - Slaughterhouse	Plateau, Nigéria	Africa	Western Africa	Aw	Abiayi et al., 2024
KR030152.1	*L. interrogans*	Nigeria-PLA11/2013	16S rRNA	Copenhageni/Icterohaemorrhagiae	Isolate	Urine	ND - Slaughterhouse	Plateau, Nigéria	Africa	Western Africa	Aw	Abiayi et al., 2024
KR030153.1	*L. interrogans*	Nigeria-FCT12/2013	16S rRNA	Copenhageni/Icterohaemorrhagiae	Isolate	Urine	ND - Slaughterhouse	Plateau, Nigéria	Africa	Western Africa	Aw	Abiayi et al., 2024
KR030154.1	*L. interrogans*	Nigeria-BNE13/2013	16S rRNA	Copenhageni/Icterohaemorrhagiae	Isolate	Urine	ND - Slaughterhouse	Plateau, Nigéria	Africa	Western Africa	Aw	Abiayi et al., 2024
KT952400.1	*L. borgpetersenii*	L49	*secY*	Hardjo/Sejroe	Isolate	Urine	Reproductive failures	Paraná, Brazil	America	South America	Csc	Chideroli et al., 2017
KT952401.1	*L. borgpetersenii*	L54	*secY*	Hardjo/Sejroe	Isolate	Urine	Reproductive failures	Paraná, Brazil	America	South America	Csc	Chideroli et al., 2017
KU183588.1	*Leptospira sp.*	BOV04	16S rRNA	-	Clinical sample	Kidney	ND	ND, La Réunion	Africa	Eastern Africa	Af	Guernier et al., 2016
KU183599.1	*Leptospira sp.*	AD212	*secY + adk*	-	Clinical sample	Kidney	ND	ND, La Réunion	Africa	Eastern Africa	Af	Guernier et al., 2016
KU183600.1	*Leptospira sp.*	AD213	*secY*	-	Clinical sample	Kidney	ND	ND, La Réunion	Africa	Eastern Africa	Af	Guernier et al., 2016
KU183601.1	*Leptospira sp.*	AD277	*secY + adk*	-	Clinical sample	Kidney	ND	ND, La Réunion	Africa	Eastern Africa	Af	Guernier et al., 2016

Table S1 - Full metadata of bovine *Leptospira* sequences and genomes deposited in GenBank and BIGSdb-Pasteur

KU183602.1	*Leptospira sp.*	AD288	*secY* + *lipL32* + *adk*	-	Clinical sample	Kidney	ND	ND, La Réunion	Africa	Eastern Africa	Af	Guernier et al., 2016
KU183603.1	*Leptospira sp.*	BOV02	*secY* + 16S rRNA + *adk*	-	Clinical sample	Kidney	ND	ND, La Réunion	Africa	Eastern Africa	Af	Guernier et al., 2016
KU183604.1	*Leptospira sp.*	BOV021	*secY*	-	Clinical sample	Kidney	ND	ND, La Réunion	Africa	Eastern Africa	Af	Guernier et al., 2016
KU216749.1/ JQ765632.1	*L. interrogans*	Lagoa	*secY* + 16S rRNA	Hardjo/Sejroe	Isolate	Urine	Reproductive failures	Minas Gerais, Brazil	America	South America	Aw	Cosate et al., 2017
KU216750.1/ JQ765630.1	*L. interrogans*	Bolívia	*secY* + 16S rRNA	Hardjo/Sejroe	Isolate	Urine	Reproductive failures	Minas Gerais, Brazil	America	South America	Aw	Cosate et al., 2017
KU219481.1	*L. interrogans*	13843	*secY* + MLST scheme #1	ND/Pomona	Isolate	ND	ND	Palmela, Portugal	Europe	Southern Europe	Csa	Ferreira et al., 2019
KU219485.1	*L. borgpetersenii*	102A	*secY* + MLST scheme #1	Hardjo/Sejroe	Isolate	ND	ND	Azores, Portugal	Europe	Southern Europe	Cfa	Ferreira et al., 2019
KU219486.1	*L. borgpetersenii*	105A	*secY* + MLST scheme #1	Hardjo/Sejroe	Isolate	ND	ND	Azores, Portugal	Europe	Southern Europe	Cfa	Ferreira et al., 2019
KU219572.1	*L. borgpetersenii*	8A	*secY* + MLST scheme #1	Hardjo/Sejroe	Isolate	ND	ND	Azores, Portugal	Europe	Southern Europe	Cfa	Ferreira et al., 2019
KU219573.1	*L. borgpetersenii*	16A	*secY* + MLST scheme #1	Hardjo/Sejroe	Isolate	ND	ND	Azores, Portugal	Europe	Southern Europe	Cfa	Ferreira et al., 2019
KU219574.1	*L. borgpetersenii*	54A	*secY* + MLST scheme #1	Hardjo/Sejroe	Isolate	ND	ND	Azores, Portugal	Europe	Southern Europe	Cfa	Ferreira et al., 2019
KU219576.1	*L. borgpetersenii*	71A	*secY* + MLST scheme #1	Hardjo/Sejroe	Isolate	ND	ND	Azores, Portugal	Europe	Southern Europe	Cfa	Ferreira et al., 2019
KU219578.1	*L. borgpetersenii*	214A	*secY* + MLST scheme #1	Hardjo/Sejroe	Isolate	ND	ND	Azores, Portugal	Europe	Southern Europe	Cfa	Ferreira et al., 2019
KU219579.1	*L. borgpetersenii*	216A	*secY* + MLST scheme #1	Hardjo/Sejroe	Isolate	ND	ND	Azores, Portugal	Europe	Southern Europe	Cfa	Ferreira et al., 2019
KU219580.1	*L. borgpetersenii*	62B	*secY* + MLST scheme #1	Hardjo/Sejroe	Isolate	ND	ND	Azores, Portugal	Europe	Southern Europe	Cfa	Ferreira et al., 2019
KU219581.1	*L. borgpetersenii*	227B	*secY* + MLST scheme #1	Hardjo/Sejroe	Isolate	ND	ND	Azores, Portugal	Europe	Southern Europe	Cfa	Ferreira et al., 2019

Table S1 - Full metadata of bovine *Leptospira* sequences and genomes deposited in GenBank and BIGSdb-Pasteur

KX712246.1	*L. interrogans*	BiCCCOH4-76	16S rRNA (*rrs*)	-	Clinical sample	Urine	ND	ND, Colombia	America	South America	ND	Ensuncho-Hoyos et al., 2017
KX712247.1	*L. interrogans*	BiCCCOH2-33	16S rRNA (*rrs*)	-	Clinical sample	Urine	ND	ND, Colombia	America	South America	ND	Ensuncho-Hoyos et al., 2017
KX712248.1	*L. interrogans*	BiCCCOH7-162	16S rRNA (*rrs*)	-	Clinical sample	Urine	ND	ND, Colombia	America	South America	ND	Ensuncho-Hoyos et al., 2017
KY113317.1	*L. noguchii*	U79/2014	*secY* + MLST scheme #1	ND/Panama	Isolate	Urine	ND - Slaughterhouse	Rio de Janeiro, Brazil	America	South America	Aw	Loureiro et al., 2020
KY113318.1	*L. noguchii*	U93/2014	*secY* + MLST scheme #1	ND/Australis	Isolate	Urine	ND - Slaughterhouse	Rio de Janeiro, Brazil	America	South America	Aw	Loureiro et al., 2020
KY113319.1	*L. noguchii*	U65/2014	*secY* + MLST scheme #1	ND/Australis	Isolate	Urine	ND - Slaughterhouse	Rio de Janeiro, Brazil	America	South America	Aw	Loureiro et al., 2020
KY113320.1	*L. noguchii*	U289/2015	*secY* + MLST scheme #1	ND/Panama	Isolate	Urine	ND - Slaughterhouse	Rio de Janeiro, Brazil	America	South America	Aw	Loureiro et al., 2020
KY113321.1	*L. noguchii*	U323/2015	*secY* + MLST scheme #1	ND/Australis	Isolate	Urine	ND - Slaughterhouse	Rio de Janeiro, Brazil	America	South America	Aw	Loureiro et al., 2020
KY113322.1	*L. noguchii*	U325/2015	*secY* + MLST scheme #1	ND/Panama	Isolate	Urine	ND - Slaughterhouse	Rio de Janeiro, Brazil	America	South America	Aw	Loureiro et al., 2020
KY113323.1	*L. noguchii*	U374/2015	*secY* + MLST scheme #1	ND/Autumnalis	Isolate	Urine	ND - Slaughterhouse	Rio de Janeiro, Brazil	America	South America	Aw	Loureiro et al., 2020
KY113324.1	*L. noguchii*	U386/2016	*secY* + MLST scheme #1	ND/Pyrogenes	Isolate	Urine	ND - Slaughterhouse	Rio de Janeiro, Brazil	America	South America	Aw	Loureiro et al., 2020
KY306666.1	*Leptospira* sp.	Umuarama10	16S rRNA (*rrs*)	-	Clinical sample	Urine	ND	Paraná, Brazil	America	South America	Csc	Unpublished

Table S1 - Full metadata of bovine *Leptospira* sequences and genomes deposited in GenBank and BIGSdb-Pasteur

KY373222.1	*L. kirschneri*	2015/06066.2	*lfb1*	-	Clinical sample	Spleen (fetus)	Abortion	Wallonia, Belgium	Europe	Western Europe	Cfa	Gregóire et al., 2020
KY373223.1	*L. interrogans*	U1610225.3	*lfb1*	-	Clinical sample	Placenta	Abortion	Wallonia, Belgium	Europe	Western Europe	Cfa	Gregóire et al., 2020
KY373224.1	*L. interrogans*	15095924-B2	*lfb1*	-	Clinical sample	Kidney (fetus)	Abortion	Wallonia, Belgium	Europe	Western Europe	Cfa	Gregóire et al., 2020
KY373225.1	*L. interrogans*	2015/9915	*lfb1*	-	Clinical sample	Liver (fetus)	Abortion	Wallonia, Belgium	Europe	Western Europe	Cfa	Gregóire et al., 2020
KY373226.1	*L. interrogans*	U1610225.2	*lfb1*	-	Clinical sample	Placenta	Abortion	Wallonia, Belgium	Europe	Western Europe	Cfa	Gregóire et al., 2020
KY373227.1	*L. interrogans*	14045546-G1	*lfb1*	-	Clinical sample	Kidney (fetus)	Abortion	Wallonia, Belgium	Europe	Western Europe	Cfa	Gregóire et al., 2020
KY373228.1	*L. interrogans*	2014/17819	*lfb1*	-	Clinical sample	Liver (fetus)	Abortion	Wallonia, Belgium	Europe	Western Europe	Cfa	Gregóire et al., 2020
KY373229.1	*L. kirschneri*	14044550-H1	*lfb1*	-	Clinical sample	Spleen (fetus)	Abortion	Wallonia, Belgium	Europe	Western Europe	Cfa	Gregóire et al., 2020
MF187959.1	*L. interrogans*	BUT46	*secY*	-	Clinical sample	Uterus	ND - Slaughterhouse	Rio de Janeiro, Brazil	America	South America	Aw	Pires et al., 2018
MF955862.1	*L. borgpetersenii*	C0025	*secY*	-	Clinical sample	Kidney	ND - Slaughterhouse	Manyara, Tanzania	Africa	Eastern Africa	Aw	Allan et al., 2018
MF955863.1	*L. borgpetersenii*	C0051	*secY*	-	Clinical sample	Kidney	ND - Slaughterhouse	Manyara, Tanzania	Africa	Eastern Africa	Aw	Allan et al., 2018
MF955864.1	*L. kirschneri*	C0059	*secY*	-	Clinical sample	Kidney	ND - Slaughterhouse	Manyara, Tanzania	Africa	Eastern Africa	Aw	Allan et al., 2018
MF955865.1	*L. borgpetersenii*	C0084	*secY*	-	Clinical sample	Kidney	ND - Slaughterhouse	Manyara, Tanzania	Africa	Eastern Africa	Aw	Allan et al., 2018
MF955867.1	*L. borgpetersenii*	C0118	*secY*	-	Clinical sample	Kidney	ND - Slaughterhouse	Manyara, Tanzania	Africa	Eastern Africa	Aw	Allan et al., 2018

Table S1 - Full metadata of bovine *Leptospira* sequences and genomes deposited in GenBank and BIGSdb-Pasteur

MF955868.1	*L. borgpetersenii*	C0131	*secY*	-	Clinical sample	Kidney	ND - Slaughterhouse	Manyara, Tanzania	Africa	Eastern Africa	Aw	Allan et al., 2018
MF955869.1	*L. borgpetersenii*	C0201	*secY*	-	Clinical sample	Kidney	ND - Slaughterhouse	Manyara, Tanzania	Africa	Eastern Africa	Aw	Allan et al., 2018
MF955873.1	*L. borgpetersenii*	C0273	*secY*	-	Clinical sample	Kidney	ND - Slaughterhouse	Manyara, Tanzania	Africa	Eastern Africa	Aw	Allan et al., 2018
MF955874.1	*L. borgpetersenii*	C0279	*secY*	-	Clinical sample	Kidney	ND - Slaughterhouse	Manyara, Tanzania	Africa	Eastern Africa	Aw	Allan et al., 2018
MF955877.1	*L. borgpetersenii*	C0518	*secY* + *lfb1*	-	Clinical sample	Kidney	ND - Slaughterhouse	Manyara, Tanzania	Africa	Eastern Africa	Aw	Allan et al., 2018
MF955878.1	*L. borgpetersenii*	C0561	*secY* + *lfb1*	-	Clinical sample	Kidney	ND - Slaughterhouse	Manyara, Tanzania	Africa	Eastern Africa	Aw	Allan et al., 2018
MF955879.1	*L. borgpetersenii*	C0592	*secY* + *lfb1*	-	Clinical sample	Kidney	ND - Slaughterhouse	Manyara, Tanzania	Africa	Eastern Africa	Aw	Allan et al., 2018
MF955880.1	*L. borgpetersenii*	C0658	*secY*	-	Clinical sample	Kidney	ND - Slaughterhouse	Manyara, Tanzania	Africa	Eastern Africa	Aw	Allan et al., 2018
MF955881.1	*L. borgpetersenii*	C0097	*secY*	Hardjo/Sejroe	Isolate	Kidney	ND - Slaughterhouse	Manyara, Tanzania	Africa	Eastern Africa	Aw	Allan et al., 2018
MF955882.1	*L. borgpetersenii*	C0101	*secY*	Hardjo/Sejroe	Isolate	Kidney	ND - Slaughterhouse	Manyara, Tanzania	Africa	Eastern Africa	Aw	Allan et al., 2018
MH231557.1	*L. santarosai*	2014_U83	*secY* + MLST scheme #1	ND/Tarassovi	Isolate	Urine	ND - Slaughterhouse	Rio de Janeiro, Brazil	America	South America	Aw	Pinto et al., 2015

Table S1 - Full metadata of bovine *Leptospira* sequences and genomes deposited in GenBank and BIGSdb-Pasteur

MH231558.1	*L. santarosai*	2015_U291	*secY* + MLST scheme #1	ND/Grippotyphosa	Isolate	Urine	ND - Slaughterhouse	Rio de Janeiro, Brazil	America	South America	Aw	Pinto et al., 2015
MH231559.1	*L. interrogans*	2015_U349	*secY* + *ompL1* + *loa22* + *lipL32* + *ligA* + MLST scheme #1	ND	Isolate	Urine	ND - Slaughterhouse	Rio de Janeiro, Brazil	America	South America	Aw	Di Azevedo et al., 2023
MH231560.1	*L. interrogans*	2015_U376	*secY* + *ompL1* + *loa22* + *lipL32* + *ligA* + MLST scheme #1	ND	Isolate	Urine	ND - Slaughterhouse	Rio de Janeiro, Brazil	America	South America	Aw	Di Azevedo et al., 2023
MH325390.1/ MH329309.1	*L. borgpetersenii*	IP1509005	*secY* + 16S rRNA	Hardjo/Sejroe	Isolate	Urine	ND - Slaughterhouse	Salto, Uruguay	America	South America	Cfa	Zarantonelli et al., 2018
MH325391.1/ MH329310.1	*L. borgpetersenii*	IP1509006	*secY* + 16S rRNA	Hardjo/Sejroe	Isolate	Urine	ND - Slaughterhouse	Salto, Uruguay	America	South America	Cfa	Zarantonelli et al., 2018
MH325392.1/ MH329312.1	*L. interrogans*	IP1509008	*secY* + 16S rRNA	Kennewicki/Pomona	Isolate	Urine	ND - Slaughterhouse	Canelones, Uruguay	America	South America	Csc	Zarantonelli et al., 2018
MH325393.1/ MH329311.1	*L. interrogans*	IP1509009	*secY* + 16S rRNA	Kennewicki/Pomona	Isolate	Urine	ND - Slaughterhouse	Canelones, Uruguay	America	South America	Csc	Zarantonelli et al., 2018
MH325394.1/ MH329313.1	*L. interrogans*	IP1509010	*secY* + 16S rRNA	Kennewicki/Pomona	Isolate	Urine	ND - Slaughterhouse	Artigas, Uruguay	America	South America	Csc	Zarantonelli et al., 2018
MH325395.1/ MH329314.1	*L. interrogans*	IP1512011	*secY* + 16S rRNA	Kennewicki/Pomona	Isolate	Urine	ND - Slaughterhouse	Paysandú, Uruguay	America	South America	Csc	Zarantonelli et al., 2018
MH325396.1/ MH329315.1	*L. borgpetersenii*	IP1512013	*secY* + 16S rRNA	Hardjo/Sejroe	Isolate	Urine	ND - Slaughterhouse	Salto, Uruguay	America	South America	Cfa	Zarantonelli et al., 2018

Table S1 - Full metadata of bovine *Leptospira* sequences and genomes deposited in GenBank and BIGSdb-Pasteur

MH325401.1/ MH329320.1	*L. interrogans*	IP1603018	*secY* + 16S rRNA	Kennewicki/Pomona	Isolate	Urine	ND - Slaughterhouse	Artigas, Uruguay	America	South America	Csc	Zarantonelli et al., 2018
MH325402.1/ MH329321.1	*L. borgpetersenii*	IP1605020	*secY* + 16S rRNA	Hardjo/Sejroe	Isolate	Urine	ND - Slaughterhouse	Canelones, Uruguay	America	South America	Csc	Zarantonelli et al., 2018
MH325404.1/ MH329323.1	*L. interrogans*	IP1609022	*secY* + 16S rRNA	Kennewicki/Pomona	Isolate	Urine	ND - Slaughterhouse	Artigas, Uruguay	America	South America	Csc	Zarantonelli et al., 2018
MH325405.1/ MH329324.1	*L. interrogans*	IP1610023	*secY* + 16S rRNA	Kennewicki/Pomona	Isolate	Urine	ND - Slaughterhouse	Lavalleja, Uruguay	America	South America	Csc	Zarantonelli et al., 2018
MH325407.1/ MH329326.1	*L. noguchii*	IP1611025	*secY* + 16S rRNA	ND/Autumnalis	Isolate	Urine	ND - Slaughterhouse	Paysandú, Uruguay	America	South America	Csc	Zarantonelli et al., 2018
MH325408.1/ MH329327.1	*L. interrogans*	IP1611026	*secY* + 16S rRNA	Kennewicki/Pomona	Isolate	Urine	ND - Slaughterhouse	Paysandú, Uruguay	America	South America	Csc	Zarantonelli et al., 2018
MH325410.1/ MH329329.1	*L. interrogans*	IP1703028	*secY* + 16S rRNA	Kennewicki/Pomona	Isolate	Urine	ND - Slaughterhouse	Paysandú, Uruguay	America	South America	Csc	Zarantonelli et al., 2018
MH325411.1/ MH329330.1	*L. interrogans*	IP1703029	*secY* + 16S rRNA	Kennewicki/Pomona	Isolate	Kidney	ND - Slaughterhouse	Paysandú, Uruguay	America	South America	Csc	Zarantonelli et al., 2018
MH325412.1/ MH329331.1	*L. borgpetersenii*	IP1704030	*secY* + 16S rRNA	Hardjo/Sejroe	Isolate	Urine	ND - Slaughterhouse	Treinta y Tres, Uruguay	America	South America	Csc	Zarantonelli et al., 2018
MH325413.1/ MH329332.1	*L. borgpetersenii*	IP1704031	*secY* + 16S rRNA	Hardjo/Sejroe	Isolate	Urine	ND - Slaughterhouse	Treinta y Tres, Uruguay	America	South America	Csc	Zarantonelli et al., 2018
MH325415.1/ MH329334.1	*L. borgpetersenii*	IP1708034	*secY* + 16S rRNA	Hardjo/Sejroe	Isolate	Urine	ND - Slaughterhouse	Soriano, Uruguay	America	South America	Csc	Zarantonelli et al., 2018

Table S1 - Full metadata of bovine *Leptospira* sequences and genomes deposited in GenBank and BIGSdb-Pasteur

MH325416.1/ MH329335.1	*L. noguchii*	IP1708035	*secY* + 16S rRNA	ND/Autumnalis	Isolate	Kidney	ND - Slaughterhouse	Rocha, Uruguay	America	South America	Cfa	Zarantonelli et al., 2018
MH325417.1/ MH329336.1	*L. borgpetersenii*	IP1708036	*secY* + 16S rRNA	Hardjo/Sejroe	Isolate	Kidney	ND - Slaughterhouse	San José, Uruguay	America	South America	Cfa	Zarantonelli et al., 2018
MH325419.1/ MH329338.1	*L. borgpetersenii*	IP1709038	*secY* + 16S rRNA	Hardjo/Sejroe	Isolate	Kidney	ND - Slaughterhouse	Cerro Largo, Uruguay	America	South America	Csc	Zarantonelli et al., 2018
MH325420.1/ MH329339.1	*L. interrogans*	IP1710039	*secY* + 16S rRNA	Kennewicki/Pomona	Isolate	Urine	ND - Slaughterhouse	Artigas, Uruguay	America	South America	Csc	Zarantonelli et al., 2018
MH325421.1/ MH329340.1	*L. interrogans*	IP1710040	*secY* + 16S rRNA	Kennewicki/Pomona	Isolate	Urine	ND - Slaughterhouse	Artigas, Uruguay	America	South America	Csc	Zarantonelli et al., 2018
MH325422.1/ MH329341.1	*L. interrogans*	IP1710043	*secY* + 16S rRNA	Kennewicki/Pomona	Isolate	Urine	ND - Slaughterhouse	Artigas, Uruguay	America	South America	Csc	Zarantonelli et al., 2018
MH325423.1/ MH329342.1	*L. interrogans*	IP1710044	*secY* + 16S rRNA	Kennewicki/Pomona	Isolate	Urine	ND - Slaughterhouse	Artigas, Uruguay	America	South America	Csc	Zarantonelli et al., 2018
MH325424.1/ MH329343.1	*L. interrogans*	IP1710045	*secY* + 16S rRNA	Kennewicki/Pomona	Isolate	Urine	ND - Slaughterhouse	Artigas, Uruguay	America	South America	Csc	Zarantonelli et al., 2018
MH325425.1/ MH329344.1	*L. interrogans*	IP1710047	*secY* + 16S rRNA	Kennewicki/Pomona	Isolate	Urine	ND - Slaughterhouse	Paysandú, Uruguay	America	South America	Csc	Zarantonelli et al., 2018
MH325426.1/ MH329345.1	*L. interrogans*	IP1711049	*secY* + 16S rRNA	Canicola/Canicola	Isolate	Kidney	ND - Slaughterhouse	Treinta y Tres, Uruguay	America	South America	Csc	Zarantonelli et al., 2018
MH683044.1	*L. kirschneri*	UFU02	*secY*	ND/Grippotyphosa	Isolate	Urine	ND - Slaughterhouse	Minas Gerais, Brazil	America	South America	Aw	Soares et al., 2020

Table S1 - Full metadata of bovine *Leptospira* sequences and genomes deposited in GenBank and BIGSdb-Pasteur

MK077114.1	*L. borgpetersenii*	169	*secY*	-	Clinical sample	Urine	ND - Slaughterhouse	Pará, Brazil	America	South America	Am	Guedes et al., 2019
MK077115.1	*L. borgpetersenii*	172	*secY*	-	Clinical sample	Urine	ND - Slaughterhouse	Pará, Brazil	America	South America	Am	Guedes et al., 2019
MK077116.1	*L. borgpetersenii*	139	*secY*	-	Clinical sample	Urine	ND - Slaughterhouse	Pará, Brazil	America	South America	Am	Guedes et al., 2019
MK077117.1	*L. borgpetersenii*	138	*secY*	-	Clinical sample	Urine	ND - Slaughterhouse	Pará, Brazil	America	South America	Am	Guedes et al., 2019
MK077118.1	*L. borgpetersenii*	130	*secY*	-	Clinical sample	Urine	ND - Slaughterhouse	Pará, Brazil	America	South America	Am	Guedes et al., 2019
MK077119.1	*L. borgpetersenii*	128u	*secY*	-	Clinical sample	Urine	ND - Slaughterhouse	Pará, Brazil	America	South America	Am	Guedes et al., 2019
MK077121.1	*L. borgpetersenii*	119u	*secY*	-	Clinical sample	Urine	ND - Slaughterhouse	Pará, Brazil	America	South America	Am	Guedes et al., 2019
MK077123.1	*L. borgpetersenii*	112	*secY*	-	Clinical sample	Urine	ND - Slaughterhouse	Pará, Brazil	America	South America	Am	Guedes et al., 2019
MK077124.1	*L. borgpetersenii*	49	*secY*	-	Clinical sample	Urine	ND - Slaughterhouse	Pará, Brazil	America	South America	Am	Guedes et al., 2019
MK077125.1	*L. borgpetersenii*	33	*secY*	-	Clinical sample	Urine	ND - Slaughterhouse	Pará, Brazil	America	South America	Am	Guedes et al., 2019
MK077126.1	*L. borgpetersenii*	35	*secY*	-	Clinical sample	Urine	ND - Slaughterhouse	Pará, Brazil	America	South America	Am	Guedes et al., 2019

Table S1 - Full metadata of bovine *Leptospira* sequences and genomes deposited in GenBank and BIGSdb-Pasteur

MK077127.1	*L. borgpetersenii*	63	*secY*	-	Clinical sample	Urine	ND - Slaughterhouse	Pará, Brazil	America	South America	Am	Guedes et al., 2019
MK077128.1	*L. borgpetersenii*	24	*secY*	-	Clinical sample	Urine	ND - Slaughterhouse	Pará, Brazil	America	South America	Am	Guedes et al., 2019
MK077129.1	*L. borgpetersenii*	106	*secY*	-	Clinical sample	Urine	ND - Slaughterhouse	Pará, Brazil	America	South America	Am	Guedes et al., 2019
MK077130.1	*L. borgpetersenii*	208	*secY*	-	Clinical sample	Urine	ND - Slaughterhouse	Pará, Brazil	America	South America	Am	Guedes et al., 2019
MK077131.1	*L. santarosai*	61	*secY*	-	Clinical sample	Urine	ND - Slaughterhouse	Pará, Brazil	America	South America	Am	Guedes et al., 2019
MK077132.1	*L. interrogans*	161	*secY*	-	Clinical sample	Urine	ND - Slaughterhouse	Pará, Brazil	America	South America	Am	Guedes et al., 2019
MK077133.1	*L. interrogans*	98	*secY*	-	Clinical sample	Urine	ND - Slaughterhouse	Pará, Brazil	America	South America	Am	Guedes et al., 2019
MK077134.1	*L. kirschneri*	42	*secY*	-	Clinical sample	Urine	ND - Slaughterhouse	Pará, Brazil	America	South America	Am	Guedes et al., 2019
MK077135.1	*L. kirschneri*	60	*secY*	-	Clinical sample	Urine	ND - Slaughterhouse	Pará, Brazil	America	South America	Am	Guedes et al., 2019
MK077136.1	*L. kirschneri*	120	*secY*	-	Clinical sample	Urine	ND - Slaughterhouse	Pará, Brazil	America	South America	Am	Guedes et al., 2019
MK077137.1	*L. kirschneri*	177	*secY*	-	Clinical sample	Urine	ND - Slaughterhouse	Pará, Brazil	America	South America	Am	Guedes et al., 2019

Table S1 - Full metadata of bovine *Leptospira* sequences and genomes deposited in GenBank and BIGSdb-Pasteur

MK077138.1	*Leptospira* sp.	30	*secY*	-	Clinical sample	Urine	ND - Slaughterhouse	Pará, Brazil	America	South America	Am	Guedes et al., 2019
MK077139.1	*Leptospira* sp.	67	*secY*	-	Clinical sample	Urine	ND - Slaughterhouse	Pará, Brazil	America	South America	Am	Guedes et al., 2019
MK077140.1	*Leptospira* sp.	105	*secY*	-	Clinical sample	Urine	ND - Slaughterhouse	Pará, Brazil	America	South America	Am	Guedes et al., 2019
MK077141.1	*Leptospira* sp.	140	*secY*	-	Clinical sample	Urine	ND - Slaughterhouse	Pará, Brazil	America	South America	Am	Guedes et al., 2019
MK077142.1	*Leptospira* sp.	157	*secY*	-	Clinical sample	Urine	ND - Slaughterhouse	Pará, Brazil	America	South America	Am	Guedes et al., 2019
MK077143.1	*Leptospira* sp.	188	*secY*	-	Clinical sample	Urine	ND - Slaughterhouse	Pará, Brazil	America	South America	Am	Guedes et al., 2019
MK244298.1	*L. kirschneri*	LZMO13u	*lfb1*	-	Clinical sample	Urine	ND - Slaughterhouse	ND, Madagascar	Africa	Eastern Africa	ND	Rahelinirina et al., 2019
MK244299.1	*L. borgpetersenii*	LZA2k	*lfb1*	-	Clinical sample	Kidney	ND - Slaughterhouse	ND, Madagascar	Africa	Eastern Africa	ND	Rahelinirina et al., 2019
MK244300.1	*L. borgpetersenii*	LZA4k	*lfb1*	-	Clinical sample	Kidney	ND - Slaughterhouse	ND, Madagascar	Africa	Eastern Africa	ND	Rahelinirina et al., 2019
MK244302.1	*L. borgpetersenii*	LZAK19u	*lfb1*	-	Clinical sample	Urine	ND - Slaughterhouse	ND, Madagascar	Africa	Eastern Africa	ND	Rahelinirina et al., 2019
MK244303.1	*L. borgpetersenii*	LZAP20u	*lfb1*	-	Clinical sample	Urine	ND - Slaughterhouse	ND, Madagascar	Africa	Eastern Africa	ND	Rahelinirina et al., 2019

Table S1 - Full metadata of bovine *Leptospira* sequences and genomes deposited in GenBank and BIGSdb-Pasteur

MK244304.1	*L. borgpetersenii*	LZAP22u	*lfb1*	-	Clinical sample	Urine	ND - Slaughterhouse	ND, Madagascar	Africa	Eastern Africa	ND	Rahelinirina et al., 2019
MK244305.1	*L. borgpetersenii*	LZAP3k	*lfb1*	-	Clinical sample	Kidney	ND - Slaughterhouse	ND, Madagascar	Africa	Eastern Africa	ND	Rahelinirina et al., 2019
MK244306.1	*L. borgpetersenii*	LZAS16k	*lfb1*	-	Clinical sample	Kidney	ND - Slaughterhouse	ND, Madagascar	Africa	Eastern Africa	ND	Rahelinirina et al., 2019
MK244308.1	*L. borgpetersenii*	LZAS21u	*lfb1*	-	Clinical sample	Urine	ND - Slaughterhouse	ND, Madagascar	Africa	Eastern Africa	ND	Rahelinirina et al., 2019
MK244309.1	*L. borgpetersenii*	LZAS23u	*lfb1*	-	Clinical sample	Urine	ND - Slaughterhouse	ND, Madagascar	Africa	Eastern Africa	ND	Rahelinirina et al., 2019
MK244310.1	*L. borgpetersenii*	LZAS5u	*lfb1*	-	Clinical sample	Urine	ND - Slaughterhouse	ND, Madagascar	Africa	Eastern Africa	ND	Rahelinirina et al., 2019
MK244311.1	*L. borgpetersenii*	LZAS6k	*lfb1*	-	Clinical sample	Kidney	ND - Slaughterhouse	ND, Madagascar	Africa	Eastern Africa	ND	Rahelinirina et al., 2019
MK244312.1	*L. borgpetersenii*	LZMO13u	*lfb1*	-	Clinical sample	Urine	ND - Slaughterhouse	ND, Madagascar	Africa	Eastern Africa	ND	Rahelinirina et al., 2019
MK244314.1	*L. borgpetersenii*	C0025k	*lfb1*	-	Clinical sample	Kidney	ND - Slaughterhouse	ND, Tanzania	Africa	Eastern Africa	ND	Rahelinirina et al., 2019
MK244315.1	*L. borgpetersenii*	C0084k	*lfb1*	-	Clinical sample	Kidney	ND - Slaughterhouse	ND, Tanzania	Africa	Eastern Africa	ND	Rahelinirina et al., 2019
MK244316.1	*L. borgpetersenii*	C0097k	*lfb1*	-	Clinical sample	Kidney	ND - Slaughterhouse	ND, Tanzania	Africa	Eastern Africa	ND	Rahelinirina et al., 2019

Table S1 - Full metadata of bovine *Leptospira* sequences and genomes deposited in GenBank and BIGSdb-Pasteur

MK244317.1	*L. borgpetersenii*	C0101k	*lfb1*	-	Clinical sample	Kidney	ND - Slaughterhouse	ND, Tanzania	Africa	Eastern Africa	ND	Rahelinirina et al., 2019
MK244318.1	*L. borgpetersenii*	C0131k	*lfb1*	-	Clinical sample	Kidney	ND - Slaughterhouse	ND, Tanzania	Africa	Eastern Africa	ND	Rahelinirina et al., 2019
MK244319.1	*L. borgpetersenii*	C0201k	*lfb1*	-	Clinical sample	Kidney	ND - Slaughterhouse	ND, Tanzania	Africa	Eastern Africa	ND	Rahelinirina et al., 2019
MK244320.1	*L. borgpetersenii*	C0273k	*lfb1*	-	Clinical sample	Kidney	ND - Slaughterhouse	ND, Tanzania	Africa	Eastern Africa	ND	Rahelinirina et al., 2019
MK244321.1	*L. borgpetersenii*	C0279k	*lfb1*	-	Clinical sample	Kidney	ND - Slaughterhouse	ND, Tanzania	Africa	Eastern Africa	ND	Rahelinirina et al., 2019
MK244322.1	*L. kirschneri*	C0059k	*lfb1*	-	Clinical sample	Kidney	ND - Slaughterhouse	ND, Tanzania	Africa	Eastern Africa	ND	Rahelinirina et al., 2019
MK244327.1	*L. borgpetersenii*	C0051k	*lfb1*	-	Clinical sample	Kidney	ND - Slaughterhouse	ND, Tanzania	Africa	Eastern Africa	ND	Rahelinirina et al., 2019
MK244328.1	*L. borgpetersenii*	C0089k	*lfb1*	-	Clinical sample	Kidney	ND - Slaughterhouse	ND, Tanzania	Africa	Eastern Africa	ND	Rahelinirina et al., 2019
MK244329.1	*L. borgpetersenii*	C0109k	*lfb1*	-	Clinical sample	Kidney	ND - Slaughterhouse	ND, Tanzania	Africa	Eastern Africa	ND	Rahelinirina et al., 2019
MK244330.1	*L. borgpetersenii*	C0118k	*lfb1*	-	Clinical sample	Kidney	ND - Slaughterhouse	ND, Tanzania	Africa	Eastern Africa	ND	Rahelinirina et al., 2019
MK244331.1	*L. borgpetersenii*	C0180k	*lfb1*	-	Clinical sample	Kidney	ND - Slaughterhouse	ND, Tanzania	Africa	Eastern Africa	ND	Rahelinirina et al., 2019

Table S1 - Full metadata of bovine *Leptospira* sequences and genomes deposited in GenBank and BIGSdb-Pasteur

MK244332.1	*L. borgpetersenii*	C0190k	*lfb1*	-	Clinical sample	Kidney	ND - Slaughterhouse	ND, Tanzania	Africa	Eastern Africa	ND	Rahelinirina et al., 2019
MK244333.1	*L. borgpetersenii*	C0195k	*lfb1*	-	Clinical sample	Kidney	ND - Slaughterhouse	ND, Tanzania	Africa	Eastern Africa	ND	Rahelinirina et al., 2019
MK244335.1	*L. borgpetersenii*	C0552k	*lfb1*	-	Clinical sample	Kidney	ND - Slaughterhouse	ND, Tanzania	Africa	Eastern Africa	ND	Rahelinirina et al., 2019
MK244338.1	*L. borgpetersenii*	C0615k	*lfb1*	-	Clinical sample	Kidney	ND - Slaughterhouse	ND, Tanzania	Africa	Eastern Africa	ND	Rahelinirina et al., 2019
MK244339.1	*L. borgpetersenii*	C0620k	*lfb1*	-	Clinical sample	Kidney	ND - Slaughterhouse	ND, Tanzania	Africa	Eastern Africa	ND	Rahelinirina et al., 2019
MK244340.1	*L. borgpetersenii*	C0633k	*lfb1*	-	Clinical sample	Kidney	ND - Slaughterhouse	ND, Tanzania	Africa	Eastern Africa	ND	Rahelinirina et al., 2019
MK244341.1	*L. borgpetersenii*	C0658k	*lfb1*	-	Clinical sample	Kidney	ND - Slaughterhouse	ND, Tanzania	Africa	Eastern Africa	ND	Rahelinirina et al., 2019
MK244342.1	*L. borgpetersenii*	LZAK19u	*secY*	-	Clinical sample	Urine	ND - Slaughterhouse	ND, Madagascar	Africa	Eastern Africa	ND	Rahelinirina et al., 2019
MK244343.1	*L. borgpetersenii*	LZAP22u	*secY*	-	Clinical sample	Urine	ND - Slaughterhouse	ND, Madagascar	Africa	Eastern Africa	ND	Rahelinirina et al., 2019
MK244344.1	*L. borgpetersenii*	LZAP20u	*secY*	-	Clinical sample	Urine	ND - Slaughterhouse	ND, Madagascar	Africa	Eastern Africa	ND	Rahelinirina et al., 2019
MK433486.1	*L. santarosai*	2016_U001JF	MLST scheme #1	ND/Tarassovi	Isolate	Urine	ND - Slaughterhouse	Rio de Janeiro, Brazil	America	South America	Aw	Jaeger et al., 2019

Table S1 - Full metadata of bovine *Leptospira* sequences and genomes deposited in GenBank and BIGSdb-Pasteur

MK433487.1	*L. noguchii*	2015_U362	MLST scheme #1	ND/Australis	Isolate	Urine	ND - Slaughterhouse	Rio de Janeiro, Brazil	America	South America	Aw	Jaeger et al., 2019
MK433502.1	*L. santarosai*	2014_U213	MLST scheme #1	Not Reactive	Isolate	Urine	ND - Slaughterhouse	Rio de Janeiro, Brazil	America	South America	Aw	Jaeger et al., 2019
MK433503.1	*L. santarosai*	2014_U222	MLST scheme #1	Not Reactive	Isolate	Urine	ND - Slaughterhouse	Rio de Janeiro, Brazil	America	South America	Aw	Jaeger et al., 2019
MK433504.1	*L. santarosai*	2015_U237	MLST scheme #1	Guaricura/Sejroe	Isolate	Urine	ND - Slaughterhouse	Rio de Janeiro, Brazil	America	South America	Aw	Jaeger et al., 2019
MK433516.1	*L. santarosai*	2016_U387	MLST scheme #1	ND/Grippotyphosa	Isolate	Urine	ND - Slaughterhouse	Rio de Janeiro, Brazil	America	South America	Aw	Jaeger et al., 2019
MK537340.1	*L. interrogans*	DD2	*secY*	-	Clinical sample	Serum	ND	Paraná, Brazil	America	South America	Csc	Unpublished
MK610269.1	*L. kirschneri*	LZMO13k	*secY*	-	Clinical sample	Kidney	ND - Slaughterhouse	ND, Madagascar	Africa	Eastern Africa	ND	Rahelinirina et al., 2019
MK610270.1	*L. borgpetersenii*	LZAS23k	*secY*	-	Clinical sample	Kidney	ND - Slaughterhouse	ND, Madagascar	Africa	Eastern Africa	ND	Rahelinirina et al., 2019
MK610271.1	*L. borgpetersenii*	LZAS21k	*secY*	-	Clinical sample	Kidney	ND - Slaughterhouse	ND, Madagascar	Africa	Eastern Africa	ND	Rahelinirina et al., 2019
MK610272.1	*L. borgpetersenii*	LZA6k	*secY*	-	Clinical sample	Kidney	ND - Slaughterhouse	ND, Madagascar	Africa	Eastern Africa	ND	Rahelinirina et al., 2019
MK610273.1	*L. borgpetersenii*	LZAP3k	*secY*	-	Clinical sample	Kidney	ND - Slaughterhouse	ND, Madagascar	Africa	Eastern Africa	ND	Rahelinirina et al., 2019

Table S1 - Full metadata of bovine *Leptospira* sequences and genomes deposited in GenBank and BIGSdb-Pasteur

MK610274.1	*L. borgpetersenii*	LZAK19k	*secY*	-	Clinical sample	Kidney	ND - Slaughterhouse	ND, Madagascar	Africa	Eastern Africa	ND	Rahelinirina et al., 2019
MN148371.1	*L. borgpetersenii*	58U	*secY*	-	Clinical sample	Urine	ND - Slaughterhouse	Kampala, Uganda	Africa	Eastern Africa	Af	Alinaitwe et al., 2019
MN148373.1	*L. kirschneri*	268K	*secY*	-	Clinical sample	Kidney	ND - Slaughterhouse	Kampala, Uganda	Africa	Eastern Africa	Af	Alinaitwe et al., 2019
MN148374.1	*L. borgpetersenii*	302K	*secY*	-	Clinical sample	Kidney	ND - Slaughterhouse	Kampala, Uganda	Africa	Eastern Africa	Af	Alinaitwe et al., 2019
MN148375.1	*L. borgpetersenii*	370U	*secY*	-	Clinical sample	Urine	ND - Slaughterhouse	Kampala, Uganda	Africa	Eastern Africa	Af	Alinaitwe et al., 2019
MN718733.1	*L. interrogans*	90U/Br	*gyrB*	-	Clinical sample	Urine	ND	Santa Catarina, Brazil	America	South America	Csc	Unpublished
MN718734.1	*L. interrogans*	92U/Br	*gyrB*	-	Clinical sample	Urine	ND	Santa Catarina, Brazil	America	South America	Csc	Unpublished
MN718735.1	*L. interrogans*	95U/Br	*gyrB*	-	Clinical sample	Urine	ND	Santa Catarina, Brazil	America	South America	Csc	Unpublished
MN718736.1	*L. interrogans*	96U/Br	*gyrB*	-	Clinical sample	Urine	ND	Santa Catarina, Brazil	America	South America	Csc	Unpublished
MN718737.1	*L. interrogans*	110U/Br	*gyrB*	-	Clinical sample	Urine	ND	Santa Catarina, Brazil	America	South America	Csc	Unpublished
MT270418.1	*L. interrogans*	FF13	*secY*	-	Clinical sample	Follicular fluid	ND - Slaughterhouse	Rio de Janeiro, Brazil	America	South America	Aw	Di Azevedo et al., 2021
MT270419.1	*L. santarosai*	FF18	*secY*	-	Clinical sample	Follicular fluid	ND - Slaughterhouse	Rio de Janeiro, Brazil	America	South America	Aw	Di Azevedo et al., 2021
MT270420.1	*L. interrogans*	UF4	*secY*	-	Clinical sample	Uterus	ND - Slaughterhouse	Rio de Janeiro, Brazil	America	South America	Aw	Di Azevedo et al., 2020

Table S1 - Full metadata of bovine *Leptospira* sequences and genomes deposited in GenBank and BIGSdb-Pasteur

MT270421.1	*L. interrogans*	UF5	*secY*	-	Clinical sample	Uterus	ND - Slaughterhouse	Rio de Janeiro, Brazil	America	South America	Aw	Di Azevedo et al., 2020
MT270422.1	*L. interrogans*	UF7	*secY*	-	Clinical sample	Uterus	ND - Slaughterhouse	Rio de Janeiro, Brazil	America	South America	Aw	Di Azevedo et al., 2020
MT270423.1	*L. interrogans*	UF9	*secY*	-	Clinical sample	Uterus	ND - Slaughterhouse	Rio de Janeiro, Brazil	America	South America	Aw	Di Azevedo et al., 2020
MT270424.1	*L. interrogans*	UF16	*secY*	-	Clinical sample	Uterus	ND - Slaughterhouse	Rio de Janeiro, Brazil	America	South America	Aw	Di Azevedo et al., 2020
MT270425.1	*L. interrogans*	UF19	*secY*	-	Clinical sample	Uterus	ND - Slaughterhouse	Rio de Janeiro, Brazil	America	South America	Aw	Di Azevedo et al., 2020
MT270426.1	*L. interrogans*	UF20	*secY*	-	Clinical sample	Uterus	ND - Slaughterhouse	Rio de Janeiro, Brazil	America	South America	Aw	Di Azevedo et al., 2020
MT270427.1	*L. interrogans*	UF21	*secY*	-	Clinical sample	Uterus	ND - Slaughterhouse	Rio de Janeiro, Brazil	America	South America	Aw	Di Azevedo et al., 2020
MT270428.1	*L. interrogans*	UF24	*secY*	-	Clinical sample	Uterus	ND - Slaughterhouse	Rio de Janeiro, Brazil	America	South America	Aw	Di Azevedo et al., 2020
MT645311.1	*L. interrogans*	ND	16S rRNA	Hardjo/Sejroe	Isolate	Urine	Systemic (Hepatic failure)	Tamil Nadu, India	Asia	Southern Asia	Aw	Unpublished
MT708145.1	*L. borgpetersenii*	M02/20-06	*secY*	Hardjo/Sejroe	Isolate	Urine	ND - Slaughterhouse	Pará, Brazil	America	South America	Am	Guedes et al., 2021
MT708146.1	*L. borgpetersenii*	M02/20-09	*secY*	Hardjo/Sejroe	Isolate	Urine	ND - Slaughterhouse	Pará, Brazil	America	South America	Am	Guedes et al., 2021

Table S1 - Full metadata of bovine *Leptospira* sequences and genomes deposited in GenBank and BIGSdb-Pasteur

MT708147.1	*L. borgpetersenii*	M02/20-23	*secY*	Hardjo/Sejroe	Isolate	Urine	ND - Slaughterhouse	Pará, Brazil	America	South America	Am	Guedes et al., 2021
MT708148.1	*L. borgpetersenii*	M02/20-44	*secY*	Hardjo/Sejroe	Isolate	Urine	ND - Slaughterhouse	Pará, Brazil	America	South America	Am	Guedes et al., 2021
MT708149.1	*L. borgpetersenii*	M02/20-50	*secY*	Hardjo/Sejroe	Isolate	Urine	ND - Slaughterhouse	Pará, Brazil	America	South America	Am	Guedes et al., 2021
MT708150.1	*L. borgpetersenii*	M02/20-52	*secY*	Hardjo/Sejroe	Isolate	Urine	ND - Slaughterhouse	Pará, Brazil	America	South America	Am	Guedes et al., 2021
MT708151.1	*L. borgpetersenii*	M02/20-53	*secY*	Hardjo/Sejroe	Isolate	Urine	ND - Slaughterhouse	Pará, Brazil	America	South America	Am	Guedes et al., 2021
MT708152.1	*L. borgpetersenii*	M02/20-55	*secY*	Hardjo/Sejroe	Isolate	Urine	ND - Slaughterhouse	Pará, Brazil	America	South America	Am	Guedes et al., 2021
MT708153.1	*L. borgpetersenii*	M02/20-67	*secY*	Hardjo/Sejroe	Isolate	Urine	ND - Slaughterhouse	Pará, Brazil	America	South America	Am	Guedes et al., 2021
MT708154.1	*L. borgpetersenii*	M02/20-68	*secY*	Hardjo/Sejroe	Isolate	Urine	ND - Slaughterhouse	Pará, Brazil	America	South America	Am	Guedes et al., 2021
MT708155.1	*L. borgpetersenii*	M02/20-77	*secY*	Hardjo/Sejroe	Isolate	Urine	ND - Slaughterhouse	Pará, Brazil	America	South America	Am	Guedes et al., 2021
MT708156.1	*L. borgpetersenii*	M02/20-96	*secY*	Hardjo/Sejroe	Isolate	Urine	ND - Slaughterhouse	Pará, Brazil	America	South America	Am	Guedes et al., 2021
MT708157.1	*L. borgpetersenii*	M02/20-116	*secY*	Hardjo/Sejroe	Isolate	Urine	ND - Slaughterhouse	Pará, Brazil	America	South America	Am	Guedes et al., 2021

Table S1 - Full metadata of bovine *Leptospira* sequences and genomes deposited in GenBank and BIGSdb-Pasteur

MT708158.1	*L. borgpetersenii*	M02/20-118	*secY*	Hardjo/Sejroe	Isolate	Urine	ND - Slaughterhouse	Pará, Brazil	America	South America	Am	Guedes et al., 2021
MT708159.1	*L. borgpetersenii*	M02/20-121	*secY*	Hardjo/Sejroe	Isolate	Urine	ND - Slaughterhouse	Pará, Brazil	America	South America	Am	Guedes et al., 2021
MT708160.1	*L. borgpetersenii*	M02/20-143	*secY*	Hardjo/Sejroe	Isolate	Urine	ND - Slaughterhouse	Pará, Brazil	America	South America	Am	Guedes et al., 2021
MT708161.1	*L. borgpetersenii*	M02/20-152	*secY*	Hardjo/Sejroe	Isolate	Urine	ND - Slaughterhouse	Pará, Brazil	America	South America	Am	Guedes et al., 2021
MT708162.1	*L. borgpetersenii*	M02/20-162	*secY*	Hardjo/Sejroe	Isolate	Urine	ND - Slaughterhouse	Pará, Brazil	America	South America	Am	Guedes et al., 2021
MT708163.1	*L. borgpetersenii*	M02/20-166	*secY*	Hardjo/Sejroe	Isolate	Urine	ND - Slaughterhouse	Pará, Brazil	America	South America	Am	Guedes et al., 2021
MT708164.1	*L. borgpetersenii*	M02/20-172	*secY*	Hardjo/Sejroe	Isolate	Urine	ND - Slaughterhouse	Pará, Brazil	America	South America	Am	Guedes et al., 2021
MT708165.1	*L. kirschneri*	M02/20-11	*secY*	ND/Grippotyp hosa	Isolate	Urine	ND - Slaughterhouse	Pará, Brazil	America	South America	Am	Guedes et al., 2021
MT708166.1	*L. kirschneri*	M02/20-22	*secY*	ND/Icterohae morrhagiae	Isolate	Urine	ND - Slaughterhouse	Pará, Brazil	America	South America	Am	Guedes et al., 2021
MT708167.1	*L. kirschneri*	M02/20-29	*secY*	ND/Autumnali s	Isolate	Urine	ND - Slaughterhouse	Pará, Brazil	America	South America	Am	Guedes et al., 2021
MT708168.1	*L. kirschneri*	M02/20-31	*secY*	ND/Icterohae morrhagiae	Isolate	Urine	ND - Slaughterhouse	Pará, Brazil	America	South America	Am	Guedes et al., 2021

Table S1 - Full metadata of bovine *Leptospira* sequences and genomes deposited in GenBank and BIGSdb-Pasteur

MT708169.1	*L. kirschneri*	M02/20-32	*secY*	ND/Icterohae morrhagiae	Isolate	Urine	ND - Slaughterhouse	Pará, Brazil	America	South America	Am	Guedes et al., 2021
MT708170.1	*L. kirschneri*	M02/20-38	*secY*	ND/Grippotyp hosa	Isolate	Urine	ND - Slaughterhouse	Pará, Brazil	America	South America	Am	Guedes et al., 2021
MT708171.1	*L. kirschneri*	M02/20-47	*secY*	ND/Icterohae morrhagiae	Isolate	Urine	ND - Slaughterhouse	Pará, Brazil	America	South America	Am	Guedes et al., 2021
MT708172.1	*L. kirschneri*	M02/20-102	*secY*	ND/Icterohae morrhagiae	Isolate	Urine	ND - Slaughterhouse	Pará, Brazil	America	South America	Am	Guedes et al., 2021
MT708173.1	*L. kirschneri*	M02/20-104	*secY*	ND/Grippotyp hosa	Isolate	Urine	ND - Slaughterhouse	Pará, Brazil	America	South America	Am	Guedes et al., 2021
MT708174.1	*L. kirschneri*	M02/20-105	*secY*	ND/Icterohae morrhagiae	Isolate	Urine	ND - Slaughterhouse	Pará, Brazil	America	South America	Am	Guedes et al., 2021
MT708175.1	*L. kirschneri*	M02/20-108	*secY*	ND/Icterohae morrhagiae	Isolate	Urine	ND - Slaughterhouse	Pará, Brazil	America	South America	Am	Guedes et al., 2021
MT708176.1	*L. kirschneri*	M02/20-111	*secY*	ND/Icterohae morrhagiae	Isolate	Urine	ND - Slaughterhouse	Pará, Brazil	America	South America	Am	Guedes et al., 2021
MT708177.1	*L. kirschneri*	M02/20-126	*secY*	ND/Grippotyp hosa	Isolate	Urine	ND - Slaughterhouse	Pará, Brazil	America	South America	Am	Guedes et al., 2021
MT708178.1	*L. kirschneri*	M02/20-137	*secY*	ND/Grippotyp hosa	Isolate	Urine	ND - Slaughterhouse	Pará, Brazil	America	South America	Am	Guedes et al., 2021
MT708179.1	*L. kirschneri*	M02/20-144	*secY*	ND/Autumnali s	Isolate	Urine	ND - Slaughterhouse	Pará, Brazil	America	South America	Am	Guedes et al., 2021

Table S1 - Full metadata of bovine *Leptospira* sequences and genomes deposited in GenBank and BIGSdb-Pasteur

MT708180.1	*L. kirschneri*	M02/20-170	*secY*	ND/Grippotyp hosa	Isolate	Urine	ND - Slaughterhouse	Pará, Brazil	America	South America	Am	Guedes et al., 2021
MT708181.1	*L. kirschneri*	M02/20-198	*secY*	ND/Icterohae morrhagiae	Isolate	Urine	ND - Slaughterhouse	Pará, Brazil	America	South America	Am	Guedes et al., 2021
MT708182.1	*L. kirschneri*	M02/20-215	*secY*	ND/Icterohae morrhagiae	Isolate	Urine	ND - Slaughterhouse	Pará, Brazil	America	South America	Am	Guedes et al., 2021
MT708183.1	*L. santarosai*	M02/20-03	*secY*	ND/Autumnali s	Isolate	Urine	ND - Slaughterhouse	Pará, Brazil	America	South America	Am	Guedes et al., 2021
MT708184.1	*L. santarosai*	M02/20-08	*secY*	ND	Isolate	Urine	ND - Slaughterhouse	Pará, Brazil	America	South America	Am	Guedes et al., 2021
MT708185.1	*L. santarosai*	M02/20-10	*secY*	ND	Isolate	Urine	ND - Slaughterhouse	Pará, Brazil	America	South America	Am	Guedes et al., 2021
MT708186.1	*L. santarosai*	M02/20-18	*secY*	ND/Tarassovi	Isolate	Urine	ND - Slaughterhouse	Pará, Brazil	America	South America	Am	Guedes et al., 2021
MT708187.1	*L. santarosai*	M02/20-84	*secY*	ND/Pyrogenes	Isolate	Urine	ND - Slaughterhouse	Pará, Brazil	America	South America	Am	Guedes et al., 2021
MT708188.1	*L. santarosai*	M02/20-114	*secY*	ND	Isolate	Urine	ND - Slaughterhouse	Pará, Brazil	America	South America	Am	Guedes et al., 2021
MT708189.1	*L. santarosai*	M02/20-115	*secY*	ND/Autumnali s	Isolate	Urine	ND - Slaughterhouse	Pará, Brazil	America	South America	Am	Guedes et al., 2021
MT708190.1	*L. santarosai*	M02/20-119	*secY*	ND	Isolate	Urine	ND - Slaughterhouse	Pará, Brazil	America	South America	Am	Guedes et al., 2021

Table S1 - Full metadata of bovine *Leptospira* sequences and genomes deposited in GenBank and BIGSdb-Pasteur

MT708191.1	*L. santarosai*	M02/20-207	*secY*	ND	Isolate	Urine	ND - Slaughterhouse	Pará, Brazil	America	South America	Am	Guedes et al., 2021
MT708192.1	*L. noguchii*	M02/20-17	*secY*	ND/Bataviae	Isolate	Urine	ND - Slaughterhouse	Pará, Brazil	America	South America	Am	Guedes et al., 2021
MT708193.1	*L. noguchii*	M02/20-35	*secY*	ND/Bataviae	Isolate	Urine	ND - Slaughterhouse	Pará, Brazil	America	South America	Am	Guedes et al., 2021
MT708194.1	*L. noguchii*	M02/20-46	*secY*	ND/Panama	Isolate	Urine	ND - Slaughterhouse	Pará, Brazil	America	South America	Am	Guedes et al., 2021
MT708195.1	*L. interrogans*	M02/20-136	*secY*	ND/Icterohae morrhagiae	Isolate	Urine	ND - Slaughterhouse	Pará, Brazil	America	South America	Am	Guedes et al., 2021
MT708196.1	*L. interrogans*	M02/20-155	*secY*	Canicola/Canic ola	Isolate	Urine	ND - Slaughterhouse	Pará, Brazil	America	South America	Am	Guedes et al., 2021
MZ044899.1	*L. interrogans*	Tanuvas 202	16S rRNA	ND/Hebdomad is	Isolate	Blood	ND	Tamil Nadu, India	Asia	Southern Asia	Aw	Unpublished
MZ099639.1	*L. interrogans*	CVM1	*secY*	-	Clinical sample	Cervicovaginal mucus	Reproductive failures	Rio de Janeiro, Brazil	America	South America	Aw	Aymée et al., 2021
MZ099640.1	*L. interrogans*	CVM2	*secY*	-	Clinical sample	Cervicovaginal mucus	Reproductive failures	Rio de Janeiro, Brazil	America	South America	Aw	Aymée et al., 2021
MZ099641.1	*L. interrogans*	UF3	*secY*	-	Clinical sample	Uterus	Reproductive failures	Rio de Janeiro, Brazil	America	South America	Aw	Aymée et al., 2021
MZ099642.1	*L. interrogans*	UF4	*secY*	-	Clinical sample	Uterus	Reproductive failures	Rio de Janeiro, Brazil	America	South America	Aw	Aymée et al., 2021
MZ099644.1	*L. noguchii*	UF10	*secY*	-	Clinical sample	Uterus	Reproductive failures	Rio de Janeiro, Brazil	America	South America	Aw	Aymée et al., 2021
MZ292511.1	*L. interrogans*	FMVZUV02	*lipL32*	-	Clinical sample	Urine	ND	Vera Cruz, Mexico	America	Central America	Aw	Unpublished

Table S1 - Full metadata of bovine *Leptospira* sequences and genomes deposited in GenBank and BIGSdb-Pasteur

MZ773519.1	*L. interrogans*	Cow037_FF	*secY*	-	Clinical sample	Follicular fluid	ND - Slaughterhouse	Rio de Janeiro, Brazil	America	South America	Aw	Pereirea et al., 2022
MZ773520.1	*L. interrogans*	Cow002_FF	*secY*	-	Clinical sample	Follicular fluid	ND - Slaughterhouse	Rio de Janeiro, Brazil	America	South America	Aw	Pereirea et al., 2022
MZ773521.1	*L. interrogans*	Cow128_FF	*secY*	-	Clinical sample	Follicular fluid	ND - Slaughterhouse	Rio de Janeiro, Brazil	America	South America	Aw	Pereirea et al., 2022
MZ773522.1	*L. interrogans*	Cow095_FF	*secY*	-	Clinical sample	Follicular fluid	ND - Slaughterhouse	Rio de Janeiro, Brazil	America	South America	Aw	Pereirea et al., 2022
MZ773523.1	*L. interrogans*	Cow038_FF	*secY*	-	Clinical sample	Follicular fluid	ND - Slaughterhouse	Rio de Janeiro, Brazil	America	South America	Aw	Pereirea et al., 2022
MZ773524.1	*L. interrogans*	Cow183_FF	*secY*	-	Clinical sample	Follicular fluid	ND - Slaughterhouse	Rio de Janeiro, Brazil	America	South America	Aw	Pereirea et al., 2022
MZ773525.1	*L. interrogans*	Cow185_FF	*secY*	-	Clinical sample	Follicular fluid	ND - Slaughterhouse	Rio de Janeiro, Brazil	America	South America	Aw	Pereirea et al., 2022
MZ773526.1	*L. interrogans*	Cow109_FF	*secY*	-	Clinical sample	Follicular fluid	ND - Slaughterhouse	Rio de Janeiro, Brazil	America	South America	Aw	Pereirea et al., 2022
MZ773527.1	*L. interrogans*	Cow114_FF	*secY*	-	Clinical sample	Follicular fluid	ND - Slaughterhouse	Rio de Janeiro, Brazil	America	South America	Aw	Pereirea et al., 2022
OK500433.1	*L. santarosai*	UT27	*secY*	Guaricura/Sejroe	Isolate	Uterus	Reproductive failures	Rio de Janeiro, Brazil	America	South America	Aw	Aymée et al., 2022b
OM993560.1	*L. borgpetersenii*	145	*sphB*	Hardjo/Sejroe	Isolate	Urethra	ND	ND, United Kingdom	Europe	Northern Europe	Cfb	Unpublished
OM993561.1	*L. borgpetersenii*	85	*sphB*	Hardjo/Sejroe	Isolate	Urine	ND	Florida, United States	America	Northern America	Csc	Unpublished

Table S1 - Full metadata of bovine *Leptospira* sequences and genomes deposited in GenBank and BIGSdb-Pasteur

OM993562.1	*L. borgpetersenii*	117	*sphB*	Hardjo/Sejroe	Isolate	Urine	ND	Florida, United States	America	Northern America	Csc	Unpublished
OM993563.1	*L. borgpetersenii*	45	*sphB*	Hardjo/Sejroe	Isolate	Kidney	ND	Iowa, United States	America	Northern America	Dfa	Unpublished
ON209505.1	*Leptospira* sp.	12	16s rRNA	-	Clinical sample	Urine	ND	ND, Nigeria	Africa	Western Africa	ND	Unpublished
ON209506.1	*Leptospira* sp.	13	16s rRNA	-	Clinical sample	Urine	ND	ND, Nigeria	Africa	Western Africa	ND	Unpublished
OP244760.1	*L. interrogans*	CVM15	*secY*	-	Clinical sample	Cervicovaginal mucus	Reproductive failures	Rio de Janeiro, Brazil	America	South America	Aw	Aymée et al., 2022a
OP244761.1	*L. interrogans*	CVM12	*secY*	-	Clinical sample	Cervicovaginal mucus	Reproductive failures	Rio de Janeiro, Brazil	America	South America	Aw	Aymée et al., 2022a
OP244762.1	*L. interrogans*	UR43	*secY*	-	Clinical sample	Urine	Reproductive failures	Rio de Janeiro, Brazil	America	South America	Aw	Aymée et al., 2022a
OP244763.1	*L. interrogans*	UR47	*secY*	-	Clinical sample	Urine	Reproductive failures	Rio de Janeiro, Brazil	America	South America	Aw	Aymée et al., 2022a
OP244764.1	*L. interrogans*	UT44	*secY*	-	Clinical sample	Uterus	Reproductive failures	Rio de Janeiro, Brazil	America	South America	Aw	Aymée et al., 2022a
OP244765.1	*L. interrogans*	UR28	*secY*	-	Clinical sample	Urine	Reproductive failures	Rio de Janeiro, Brazil	America	South America	Aw	Aymée et al., 2022a
OP244766.1	*L. interrogans*	UR17	*secY*	-	Clinical sample	Urine	Reproductive failures	Rio de Janeiro, Brazil	America	South America	Aw	Aymée et al., 2022a
OP244767.1	*L. interrogans*	UT30	*secY*	-	Clinical sample	Uterus	Reproductive failures	Rio de Janeiro, Brazil	America	South America	Aw	Aymée et al., 2022a
OP244768.1	*L. interrogans*	UT41	*secY*	-	Clinical sample	Uterus	Reproductive failures	Rio de Janeiro, Brazil	America	South America	Aw	Aymée et al., 2022a
OP244769.1	*L. interrogans*	UT46	*secY*	-	Clinical sample	Uterus	Reproductive failures	Rio de Janeiro, Brazil	America	South America	Aw	Aymée et al., 2022a
OP244770.1	*L. interrogans*	UT48	*secY*	-	Clinical sample	Uterus	Reproductive failures	Rio de Janeiro, Brazil	America	South America	Aw	Aymée et al., 2022a
OP244771.1	*L. santarosai*	UT18	*secY*	-	Clinical sample	Uterus	Reproductive failures	Rio de Janeiro, Brazil	America	South America	Aw	Aymée et al., 2022a

Table S1 - Full metadata of bovine *Leptospira* sequences and genomes deposited in GenBank and BIGSdb-Pasteur

OP244773.1	*L. borgpetersenii*	UT47	*secY*	-	Clinical sample	Uterus	Reproductive failures	Rio de Janeiro, Brazil	America	South America	Aw	Aymée et al., 2022a
OP244773.1	*L. noguchii*	UR11	*secY*	-	Clinical sample	Urine	Reproductive failures	Rio de Janeiro, Brazil	America	South America	Aw	Aymée et al., 2022a
OP763463.1	*L. santarosai*	2014_U76	*secY* + *ompL1* + *loa22* + *lipL32* + *ligA* + MLST scheme #1	ND	Isolate	Urine	ND - Slaughterhouse	Rio de Janeiro, Brazil	America	South America	Aw	Di Azevedo et al., 2023
OP763464.1	*L. santarosai*	2014_U140	*secY* + *ompL1* + *loa22* + *lipL32* + *ligA* + MLST scheme #1	ND	Isolate	Urine	ND - Slaughterhouse	Rio de Janeiro, Brazil	America	South America	Aw	Di Azevedo et al., 2023
OP763465.1	*L. santarosai*	U214/2013	*secY* + *ompL1* + *loa22* + *lipL32* + *ligA* + MLST scheme #1	ND	Isolate	Urine	ND - Slaughterhouse	Rio de Janeiro, Brazil	America	South America	Aw	Di Azevedo et al., 2023
OP763466.1	*L. santarosai*	2014_U81	*secY* + *ompL1* + *loa22* + *lipL32* + *ligA* + MLST scheme #1	ND	Isolate	Urine	ND - Slaughterhouse	Rio de Janeiro, Brazil	America	South America	Aw	Di Azevedo et al., 2023
OP763468.1	*L. santarosai*	2014_VF66	*ompL1* + *loa22* + *lipL32* + *ligA*	ND	Isolate	Vaginal mucus	ND - Slaughterhouse	Rio de Janeiro, Brazil	America	South America	Aw	Di Azevedo et al., 2023
OP763469.1/ OP763459.1/ OP763449.1/ OP763439.1	*L. borgpetersenii*	M02/20-06	*ompL1* + *loa22* + *lipL32* + *ligA*	ND	Isolate	Urine	ND - Slaughterhouse	Pará, Brazil	America	South America	Am	Di Azevedo et al., 2023

Table S1 - Full metadata of bovine *Leptospira* sequences and genomes deposited in GenBank and BIGSdb-Pasteur

OP763470.1/ OP763460.1/ OP763450.1/ OP763440.1	*L. borgpetersenii*	M02/20-09	*ompL1* + *loa22* + *lipL32* + *ligA*	ND	Isolate	Urine	ND - Slaughterhouse	Pará, Brazil	America	South America	Am	Di Azevedo et al., 2023
OQ116737.1	*L. interrogans*	MCV56	*secY*	-	Clinical sample	Cervicovaginal mucus	Reproductive failures	Rio de Janeiro, Brazil	America	South America	Aw	Aymée et al., 2023a
OQ116738.1	*L. interrogans*	MCV62	*secY*	-	Clinical sample	Cervicovaginal mucus	Reproductive failures	Rio de Janeiro, Brazil	America	South America	Aw	Aymée et al., 2023a
OQ116739.1	*L. interrogans*	MCV63	*secY*	-	Clinical sample	Cervicovaginal mucus	Reproductive failures	Rio de Janeiro, Brazil	America	South America	Aw	Aymée et al., 2023b
OQ116740.1	*L. interrogans*	UT74	*secY*	-	Clinical sample	Uterus	Reproductive failures	Rio de Janeiro, Brazil	America	South America	Aw	Aymée et al., 2023b
OQ116741.1	*L. interrogans*	UT67	*secY*	-	Clinical sample	Uterus	Reproductive failures	Rio de Janeiro, Brazil	America	South America	Aw	Aymée et al., 2023b
OQ116742.1	*L. interrogans*	UT62	*secY*	-	Clinical sample	Uterus	Reproductive failures	Rio de Janeiro, Brazil	America	South America	Aw	Aymée et al., 2023b
OQ116743.1	*L. interrogans*	UT83	*secY*	-	Clinical sample	Uterus	Reproductive failures	Rio de Janeiro, Brazil	America	South America	Aw	Aymée et al., 2023b
OQ116744.1	*L. interrogans*	MCV24	*secY*	-	Clinical sample	Cervicovaginal mucus	Reproductive failures	Rio de Janeiro, Brazil	America	South America	Aw	Aymée et al., 2023b
OQ116745.1	*L. interrogans*	MCV72	*secY*	-	Clinical sample	Cervicovaginal mucus	Reproductive failures	Rio de Janeiro, Brazil	America	South America	Aw	Aymée et al., 2023b
OQ116746.1	*L. interrogans*	MCV104	*secY*	-	Clinical sample	Cervicovaginal mucus	Reproductive failures	Rio de Janeiro, Brazil	America	South America	Aw	Aymée et al., 2023b
OQ421810.1	*L. borgpetersenii*	intanaina_18	16S rRNA	-	Clinical sample	Kidney	ND	Kelantan, Malaysia	Asia	Southeast Asia	Af	Unpublished
OQ421811.1	*L. borgpetersenii*	intanaina_19	16S rRNA	-	Clinical sample	Kidney	ND	Kelantan, Malaysia	Asia	Southeast Asia	Af	Unpublished
OQ421812.1	*L. borgpetersenii*	intanaina_23	16S rRNA	-	Clinical sample	Kidney	ND	Kelantan, Malaysia	Asia	Southeast Asia	Af	Unpublished
OQ421813.1	*L. borgpetersenii*	intanaina_24	16S rRNA	-	Clinical sample	Kidney	ND	Kelantan, Malaysia	Asia	Southeast Asia	Af	Unpublished

Table S1 - Full metadata of bovine *Leptospira* sequences and genomes deposited in GenBank and BIGSdb-Pasteur

OQ421814.1	*L. borgpetersenii*	intanaina_26	16S rRNA	-	Clinical sample	Kidney	ND	Kelantan, Malaysia	Asia	Southeast Asia	Af	Unpublished
OQ421815.1	*L. borgpetersenii*	intanaina_36	16S rRNA	-	Clinical sample	Kidney	ND	Kelantan, Malaysia	Asia	Southeast Asia	Af	Unpublished
OQ421816.1	*L. borgpetersenii*	intanaina_39	16S rRNA	-	Clinical sample	Kidney	ND	Kelantan, Malaysia	Asia	Southeast Asia	Af	Unpublished
OQ421817.1	*L. borgpetersenii*	intanaina_45	16S rRNA	-	Clinical sample	Kidney	ND	Kelantan, Malaysia	Asia	Southeast Asia	Af	Unpublished
OQ421818.1	*L. borgpetersenii*	intanaina_46	16S rRNA	-	Clinical sample	Kidney	ND	Kelantan, Malaysia	Asia	Southeast Asia	Af	Unpublished
OQ421820.1	*L. interrogans*	intanaina_20	16S rRNA	-	Clinical sample	Kidney	ND	Kelantan, Malaysia	Asia	Southeast Asia	Af	Unpublished
OR032919.1	*Leptospira sp.*	YY184-01	*gmlU*	-	Clinical sample	Urine	ND	Bay of Plenty, New Zealand	Oceania	Australia and New Zealand	Cfa	Unpublished
OR338746.1	*L. interrogans*	UT85	*secY*	-	Clinical sample	Uterus	Reproductive failures	Rio de Janeiro, Brazil	America	South America	Aw	Aymée et al., 2023a
OR338747.1	*L. interrogans*	UT86	*secY*	-	Clinical sample	Uterus	Reproductive failures	Rio de Janeiro, Brazil	America	South America	Aw	Aymée et al., 2023a
OR338748.1	*L. interrogans*	UT87	*secY*	-	Clinical sample	Uterus	Reproductive failures	Minas Gerais, Brazil	America	South America	Cwa	Aymée et al., 2023a
OR338749.1	*L. interrogans*	UT89	*secY*	-	Clinical sample	Uterus	Reproductive failures	Minas Gerais, Brazil	America	South America	Cwa	Aymée et al., 2023a
OR338752.1	*L. interrogans*	FetusC_Liver	*secY*	-	Clinical sample	Liver (fetus)	Abortion	Minas Gerais, Brazil	America	South America	Cwa	Aymée et al., 2023a
OR360783.1	*Leptospira sp.*	Pacifica	*gyrB*	-	Clinical sample	ND	ND	ND, New Zealand	Oceania	Australia and New Zealand	Cfa	Unpublished
OR611992.1	*L. interrogans*	CVM56	*secY*	-	Clinical sample	Cervicovaginal mucus	Reproductive failures	Rio de Janeiro, Brazil	America	South America	Aw	Borges et al., 2024
OR611993.1	*L. interrogans*	CVM62	*secY*	-	Clinical sample	Cervicovaginal mucus	Reproductive failures	Rio de Janeiro, Brazil	America	South America	Aw	Borges et al., 2024

Table S1 - Full metadata of bovine *Leptospira* sequences and genomes deposited in GenBank and BIGSdb-Pasteur

OR611994.1	*L. interrogans*	UT63	*secY*	-	Clinical sample	Uterus	Reproductive failures	Rio de Janeiro, Brazil	America	South America	Aw	Borges et al., 2024
OR611995.1	*L. interrogans*	UT64	*secY*	-	Clinical sample	Uterus	Reproductive failures	Rio de Janeiro, Brazil	America	South America	Aw	Borges et al., 2024
OR611996.1	*L. interrogans*	UT67	*secY*	-	Clinical sample	Uterus	Reproductive failures	Rio de Janeiro, Brazil	America	South America	Aw	Borges et al., 2024
OR611997.1	*L. interrogans*	UT72	*secY*	-	Clinical sample	Uterus	Reproductive failures	Rio de Janeiro, Brazil	America	South America	Aw	Borges et al., 2024
OR611998.1	*L. interrogans*	UT83	*secY*	-	Clinical sample	Uterus	Reproductive failures	Rio de Janeiro, Brazil	America	South America	Aw	Borges et al., 2024
OR611999.1	*L. interrogans*	UT90	*secY*	-	Clinical sample	Uterus	Reproductive failures	Rio de Janeiro, Brazil	America	South America	Aw	Borges et al., 2024
OR612000.1	*L. interrogans*	UT100	*secY*	-	Clinical sample	Uterus	Reproductive failures	Rio de Janeiro, Brazil	America	South America	Aw	Borges et al., 2024
PP400319.1	*L. interrogans*	FMVZ-03	*lipL32*	-	Clinical sample	Urine	ND	Vera Cruz, Mexico	America	Central America	Aw	Unpublished
PP400320.1	*L. interrogans*	FMVZ-04	*lipL32*	-	Clinical sample	Urine	ND	Vera Cruz, Mexico	America	Central America	Aw	Unpublished
PP481960.1	*L. interrogans*	B19	*lipL32*	-	Clinical sample	Urine	ND	Kerala, India	Asia	Southern Asia	Am	Unpublished
PQ039544.1	*L. interrogans*	CVM7946	*secY*	-	Clinical sample	Cervicovaginal mucus	Reproductive failures	Minas Gerais, Brazil	America	South America	Cwa	Pedrosa et al., 2025a
PQ039545.1	*L. interrogans*	CVM2988	*secY*	-	Clinical sample	Cervicovaginal mucus	Reproductive failures	Minas Gerais, Brazil	America	South America	Cwa	Pedrosa et al., 2025a
PQ039546.1	*L. interrogans*	CVM9572	*secY*	-	Clinical sample	Cervicovaginal mucus	Reproductive failures	Minas Gerais, Brazil	America	South America	Cwa	Pedrosa et al., 2025a
PQ039547.1	*L. interrogans*	CVM17884	*secY*	-	Clinical sample	Cervicovaginal mucus	Reproductive failures	Minas Gerais, Brazil	America	South America	Cwa	Pedrosa et al., 2025a
PQ039548.1	*L. interrogans*	CVM2635	*secY*	-	Clinical sample	Cervicovaginal mucus	Reproductive failures	Minas Gerais, Brazil	America	South America	Cwa	Pedrosa et al., 2025a
PQ039549.1	*L. interrogans*	CVM2985	*secY*	-	Clinical sample	Cervicovaginal mucus	Reproductive failures	Minas Gerais, Brazil	America	South America	Cwa	Pedrosa et al., 2025a

Table S1 - Full metadata of bovine *Leptospira* sequences and genomes deposited in GenBank and BIGSdb-Pasteur

PQ039551.1	*L. interrogans*	CVM2710	*secY*	-	Clinical sample	Cervicovaginal mucus	Reproductive failures	Minas Gerais, Brazil	America	South America	Cwa	Pedrosa et al., 2025a
PQ306295.1	*L. interrogans*	600EP	*secY*	-	Clinical sample	Epididymis	ND - Slaughterhouse	Rio de Janeiro, Brazil	America	South America	Aw	Pedrosa et al., 2025b
PQ306296.1	*L. interrogans*	602EP	*secY*	-	Clinical sample	Epididymis	ND - Slaughterhouse	Rio de Janeiro, Brazil	America	South America	Aw	Pedrosa et al., 2025b
PQ306297.1	*L. interrogans*	676EP	*secY*	-	Clinical sample	Epididymis	ND - Slaughterhouse	Rio de Janeiro, Brazil	America	South America	Aw	Pedrosa et al., 2025b
PQ306298.1	*L. interrogans*	1155EP	*secY*	-	Clinical sample	Epididymis	ND - Slaughterhouse	Rio de Janeiro, Brazil	America	South America	Aw	Pedrosa et al., 2025b
PQ362325.1/ PQ326448.1	*L. santarosai*	Lep031	*rpoB* + 16S rRNA	-	Clinical sample	Kidney	ND	Cundinamarca, Colômbia	America	South America	Cfb	Unpublished
PQ362332.1/ PQ326455.1	*L. interrogans*	Lep038	*rpoB* + 16S rRNA	-	Clinical sample	Fetus	ND	Cundinamarca, Colômbia	America	South America	Cfb	Unpublished
PQ362333.1/ PQ326456.1	*L. interrogans*	Lep039	*rpoB* + 16S rRNA	-	Clinical sample	Fetus	ND	Cundinamarca, Colômbia	America	South America	Cfb	Unpublished
PQ362375.1/ PQ326498.1	*L. wolffii*	Lep081	*rpoB* + 16S rRNA	-	Clinical sample	Kidney	ND	Nariño, Colombia	America	South America	Cfb	Unpublished
PQ362376.1/ PQ326499.1	*L. interrogans*	Lep091	*rpoB* + 16S rRNA	-	Clinical sample	Kidney	ND	Meta, Colombia	America	South America	Am	Unpublished
PQ362377.1/ PQ326500.1	*L. wolffii*	Lep092	*rpoB* + 16S rRNA	-	Clinical sample	Urine	ND	Cundinamarca, Colômbia	America	South America	Cfb	Unpublished
PQ362378/ PQ326501.1	*L. interrogans*	Lep093	*rpoB* + 16S rRNA	-	Clinical sample	Kidney	ND	Cundinamarca, Colômbia	America	South America	Cfb	Unpublished
PQ362379/ PQ326502.1	*L. interrogans*	Lep094	*rpoB* + 16S rRNA	-	Clinical sample	Urine	ND	Meta, Colombia	America	South America	Am	Unpublished
PQ632973.1	*L. interrogans*	Cow275	*secY*	-	Clinical sample	Cervicovaginal mucus	Reproductive failures	Minas Gerais, Brazil	America	South America	Cwa	Borges et al., 2024
PQ632974.1	*L. interrogans*	Cow018	*secY*	-	Clinical sample	Cervicovaginal mucus	Reproductive failures	Minas Gerais, Brazil	America	South America	Cwa	Borges et al., 2024

Table S1 - Full metadata of bovine *Leptospira* sequences and genomes deposited in GenBank and BIGSdb-Pasteur

PQ632974.1	*L. interrogans*	Cow08	*secY*	-	Clinical sample	Cervicovaginal mucus	Reproductive failures	Minas Gerais, Brazil	America	South America	Cwa	Borges et al., 2024
PQ632975.1	*L. interrogans*	FF104	*secY*	-	Clinical sample	Follicular fluid	ND - Slaughterhouse	Rio de Janeiro, Brazil	America	South America	Aw	Borges et al., 2024
PQ632976.1	*L. interrogans*	UT002	*secY*	-	Clinical sample	Uterus	ND - Slaughterhouse	Rio de Janeiro, Brazil	America	South America	Aw	Borges et al., 2024
PQ632977.1	*L. interrogans*	UT053	*secY*	-	Clinical sample	Uterus	ND - Slaughterhouse	Rio de Janeiro, Brazil	America	South America	Aw	Borges et al., 2024
PQ632978.1	*L. interrogans*	UT289	*secY*	-	Clinical sample	Uterus	ND - Slaughterhouse	Rio de Janeiro, Brazil	America	South America	Aw	Borges et al., 2024
PQ632979.1	*L. interrogans*	FF278	*secY*	-	Clinical sample	Follicular fluid	ND - Slaughterhouse	Rio de Janeiro, Brazil	America	South America	Aw	Borges et al., 2024
PQ632980.1	*L. interrogans*	FF137	*secY*	-	Clinical sample	Follicular fluid	ND - Slaughterhouse	Rio de Janeiro, Brazil	America	South America	Aw	Borges et al., 2024
PV018838.1	*Leptospira* sp.	KURCACR001	16S rRNA	-	Clinical sample	Urine	ND	Kurunegala, Sri Lanka	Asia	Southern Asia	Am	Unpublished
PV737841.1	*L. interrogans*	TS02RT-0081	*gmlU*	-	Clinical sample	Urine	ND	ND, New Zealand	Oceania	Australia and New Zealand	Cfa	Unpublished
PV737842.1	*L. interrogans*	TS09HB-0246	*gmlU*	-	Clinical sample	Urine	ND	ND, New Zealand	Oceania	Australia and New Zealand	Cfa	Unpublished
PV737847.1	*L. borgpetersenii*	FF0010	*gmlU*	-	Clinical sample	Kidney	ND	ND, New Zealand	Oceania	Australia and New Zealand	Cfa	Nisa et al., 2026
PV737848.1	*L. borgpetersenii*	FF0011	*gmlU*	-	Clinical sample	Kidney	ND	ND, New Zealand	Oceania	Australia and New Zealand	Cfa	Nisa et al., 2026

Table S1 - Full metadata of bovine *Leptospira* sequences and genomes deposited in GenBank and BIGSdb-Pasteur

PV737849.1	*L. borgpetersenii*	FF0020	*gmlU*	-	Clinical sample	Kidney	ND	ND, New Zealand	Oceania	Australia and New Zealand	Cfa	Nisa et al., 2026
PV737855.1	*L. borgpetersenii*	TS04R2-B048	*gmlU*	-	Clinical sample	Urine	ND	ND, New Zealand	Oceania	Australia and New Zealand	Cfa	Nisa et al., 2026
PV737855.1	*L. borgpetersenii*	TS04R2-B048	*gmlU*	-	Clinical sample	Urine	ND	ND, New Zealand	Oceania	Australia and New Zealand	Cfa	Nisa et al., 2026
PV737863.1	*L. borgpetersenii*	TS04R2_B046	*gmlU*	-	Clinical sample	Urine	ND	ND, New Zealand	Oceania	Australia and New Zealand	Cfa	Nisa et al., 2026
PV737864.1	*L. borgpetersenii*	TS05RT-0282	*gmlU*	-	Clinical sample	Urine	ND	ND, New Zealand	Oceania	Australia and New Zealand	Cfa	Nisa et al., 2026
PX105710.1	*L. borgpetersenii*	PR062-V8	16S rRNA	-	Clinical sample	Urine	ND	ND, Puerto Rico	America	Caribbean	Af	Yasir et al., 2025
PX711282.1	*Leptospira* sp.	1	16S rRNA (*rrs*)	-	Clinical sample	Urine	ND	Kelantan, Malaysia	Asia	Southeast Asia	Af	Unpublished
SAMEA5168082	*L. venezuelensis*	201502610	cgMLST + 16s rRNA + *secY*	ND	Isolate	ND	ND	ND, Venezuela	America	South America	ND	Guglielmini et al., 2019
SAMEA5168160	*L. interrogans*	201700039	cgMLST + 16s rRNA + *lfb1* + *secY*	ND/Pomona	Isolate	ND	ND	ND, United States	America	Northern America	ND	Guglielmini et al., 2019
SAMEA5168165	*L. borgpetersenii*	201700040	cgMLST + 16s rRNA + *lfb1* + *secY*	Szwajizak/Mini	Isolate	ND	ND	ND, United States	America	Northern America	ND	Guglielmini et al., 2019
SAMEA5168179	*L. kirschneri*	201700045	cgMLST + 16s rRNA + *lfb1* + *secY*	ND/Grippotyphosa	Isolate	ND	ND	ND, Turkey	Asia	Western Asia	ND	Guglielmini et al., 2019
SAMEA5168180	*L. interrogans*	201700047	cgMLST + 16s rRNA + *lfb1* + *secY*	Hardjo/Sejroe	Isolate	ND	ND	ND, United Kingdom	Europe	Northern Europe	Cfb	Guglielmini et al., 2019

Table S1 - Full metadata of bovine *Leptospira* sequences and genomes deposited in GenBank and BIGSdb-Pasteur

SAMEA5168181	*L. interrogans*	201700048	cgMLST + 16s rRNA + *lfb1* + *secY*	Hardjo/Sejroe	Isolate	ND	ND	ND, Indonesia	Asia	Southeast Asia	ND	Guglielmini et al., 2019
SAMEA5168183	*L. noguchii*	201700038	cgMLST + 16s rRNA + *lfb1* + *secY*	Peruviana/Australis	Isolate	ND	ND	ND, Peru	America	South America	ND	Guglielmini et al., 2019
SAMEA5168186	*L. kirschneri*	201700037	cgMLST + 16s rRNA + *lfb1* + *secY*	Galtoni/Canicola	Isolate	ND	ND	ND, Argentina	America	South America	ND	Guglielmini et al., 2019
SAMEA5168187	*L. interrogans*	201700041	cgMLST + 16s rRNA + *lfb1* + *secY*	Kennewicki/Pomona	Isolate	ND	ND	ND, United States	America	Northern America	ND	Guglielmini et al., 2019
SAMEA5168192	*L. interrogans*	201700043	cgMLST + 16s rRNA + *lfb1* + *secY*	ND/Pomona	Isolate	Urine	ND	New Caledonia	Oceania	Melanesia	ND	Guglielmini et al., 2019
SAMEA5168212	*L. interrogans*	201700301	cgMLST + 16s rRNA + *lfb1* + *secY*	ND/Pomona	Isolate	Urine	ND	ND, Italy	Europe	Southern Europe	Csa	Guglielmini et al., 2019
SAMEA5168215	*L. interrogans*	201700316	cgMLST + 16s rRNA + *lfb1* + *secY*	ND/Pomona	Isolate	Urine	ND	ND, Italy	Europe	Southern Europe	Csa	Guglielmini et al., 2019
SAMEA5168216	*L. borgpetersenii*	201700317	cgMLST + 16s rRNA + *lfb1* + *secY*	ND/Sejroe	Isolate	Urine	ND	ND, Uruguay	America	South America	Csc	Guglielmini et al., 2019
SAMEA5168217	*L. interrogans*	201700303	cgMLST + 16s rRNA + *lfb1* + *secY*	ND/Pomona	Isolate	Urine	ND	ND, Italy	Europe	Southern Europe	Csa	Guglielmini et al., 2019
SAMEA5168218	*L. interrogans*	201700305	cgMLST + 16s rRNA + *lfb1* + *secY*	ND/Pomona	Isolate	Urine	ND	ND, Italy	Europe	Southern Europe	Csa	Guglielmini et al., 2019
SAMEA5168343	*L. interrogans*	IP1710046	cgMLST + 16s rRNA + *lfb1* + *secY*	ND	Isolate	ND	ND	ND, Uruguay	America	South America	Csc	Unpublished

Table S1 - Full metadata of bovine *Leptospira* sequences and genomes deposited in GenBank and BIGSdb-Pasteur

SAMEA5168345	*L. borgpetersenii*	IP1711048	cgMLST + 16s rRNA + *lfb1* + *secY*	ND	Isolate	ND	ND	ND, Uruguay	America	South America	Csc	Guglielmini et al., 2019
SAMN01036871	*L. santarosai*	CBC1416	cgMLST + 16s rRNA + *lfb1* + *secY*	ND	Isolate	ND	ND	ND, Peru	America	South America	ND	Guglielmini et al., 2019
SAMN01919772	*L. santarosai*	CBC1531	cgMLST + 16s rRNA + *lfb1* + *secY*	ND	Isolate	ND	ND	ND, Peru	America	South America	ND	Guglielmini et al., 2019
SAMN01919806	*L. kirschneri*	Vehlefans 2	cgMLST + 16s rRNA + *lfb1* + *secY*	Mozdok/Pomona	Isolate	ND	ND	ND, The Netherlands	Europe	Western Europe	Cfa	Guglielmini et al., 2019
SAMN01920623	*L. santarosai*	Oregon	cgMLST + 16s rRNA + *lfb1* + *secY*	Szwajizak/Mini	Isolate	ND	ND	ND, United States	America	Northern America	ND	Guglielmini et al., 2019
SAMN02436504	*L. santarosai*	CBC523	cgMLST + 16s rRNA + *lfb1* + *secY*	ND	Isolate	ND	ND	ND, Peru	America	South America	ND	Guglielmini et al., 2019
SAMN02603616	*L. borgpetersenii*	JB197	cgMLST + 16s rRNA + *lfb1* + *secY*	Hardjo/Sejroe	Isolate	ND	ND	ND, United States	America	Northern America	ND	Guglielmini et al., 2019
SAMN16295636	*L. borgpetersenii*	I53	cgMLST + *lfb1* + *secY*	Hardjo/Sejroe	Isolate	ND	ND	New Zealand	Oceania	Australia and New Zealand	Cfa	Unpublished
SAMN16295637	*L. borgpetersenii*	I89	cgMLST + *lfb1* + *secY*	ND	Isolate	ND	ND	New Zealand	Oceania	Australia and New Zealand	Cfa	Unpublished
SAMN16295651	*L. interrogans*	A_Bt_HO905	cgMLST + 16s rRNA + *lfb1* + *secY*	ND/Pomona	Isolate	ND	ND	New Zealand	Oceania	Australia and New Zealand	Cfa	Unpublished
SAMN24659830	*L. santarosai*	DCP-017	cgMLST + *lfb1* + *secY*	ND	Isolate	Urine	ND	ND, Puerto Rico	America	Caribbean	Af	Unpublished

Table S1 - Full metadata of bovine *Leptospira* sequences and genomes deposited in GenBank and BIGSdb-Pasteur

Af - Tropical fully humid (Tropical rainforest climate)

Am - Tropical monsoon climate

Aw - Tropical dry winter (Tropical savannah climate)

BSh - Hot semi-arid (steppe climate)

Cfa - Temperate fully humid hot summer (humid subtropical climate)

Cfb - Temperate fully humid warm summer (oceanic climate)

Csa - Hot-summer mediterranean climate

Csc - Temperate dry winter short summer

Cwa - Temperate dry winter hot summer (humid subtropical climate)

Cwb - Temperate dry winter warm summer (Subtropical highland climate)

Dfa - Continental/cold fully humid hot summer

ND – Not determined
