# Supplementary material for: Genetic Epidemiology of Bovine Leptospirosis: A Global Perspective from Sequence and Genome Datasets
Source: Animals (Basel). 2026 Jul 2;16(13):2017. doi: 10.3390/ani16132017 (PMC13359918; doi:10.3390/ani16132017)
Supplement: Supplementary file 1 [file animals-16-02017-s001.zip › Supplementary Table S2.pdf]

Table S2: Distribution of bovine *Leptospira* records by country

Country	Frequency of Records
Brazil	246
Uruguay	46
Tanzania	37
India	30
United States	23
Madagascar	22
Sri Lanka	21
New Zealand	17
Colombia	14
Argentina	12
Portugal	11
Malaysia	11
Belgium	10
La Reunion	7
Italy	7
Israel	7
Nigeria	6
Puerto Rico	5
United Kingdom	5
Uganda	4
Venezuela	4
Peru	4
Mexico	3
Cuba	3
Kazakhstan	2
Ecuador	2
China	2
Ireland	1
Australia	1
France	1
Indonesia	1
Iran	1
New Caledonia	1
Turkey	1
The Netherlands	1
