# Supplementary material for: Genetic Epidemiology of Bovine Leptospirosis: A Global Perspective from Sequence and Genome Datasets
Source: Animals (Basel). 2026 Jul 2;16(13):2017. doi: 10.3390/ani16132017 (PMC13359918; doi:10.3390/ani16132017)
Supplement: Supplementary file 1 [file animals-16-02017-s001.zip › Supplementary Table S3.pdf]

Table S3. Bovine-origin *Leptospira* genome and sequence records stratified into South America and other countries/continents to minimize potential misclassification biases.

Other than South America				
**Records**	**Markers**	**Species**	**Serogroup**	**Samples**
**241 records:**<br>30 WGS e 211 sequences/MLST. From that, 89 isolates (36.9%) and 72 were serogrouped	81 *secY* (38.4%);<br>77 *lfb1* (36.5%);<br>52 *rrs* (24.6%);<br>25 *rpoB* (11.8%);<br>20 *flaB* (9.5%);<br>10 *glmU* (4.7%);<br>5 *lipL32* (2.4%);<br>4 *sphB* (1.9%); and<br>2 *gyrB* (0.9%).	126 *L. borgpetersenii* (52.3%);<br>61 *L. interrogans* (25.3%);<br>11 *L. kirschneri* (4.5%);<br>3 *L. santarosai* (1.2%);<br>1 *L. weilii* (0.4%);<br>1 *L. alexanderi* (0.4%);<br>and 38 not defined (15.8%).	52.8% Sejroe;<br>47.2% non-Sejroe	173 samples from the renal tract (71.8%);<br>22 from serum or blood (9.1%);<br>12 from fetal tissues and/or placenta (5.0%);<br>and 4 from genital samples (1.6%).
Other than South America				
**Records**	**Markers**	**Species**	**Serogroup**	**Samples**
**328 records**:<br>33 WGS e 295 sequences/MLST. 168 isolates (51.2%), 134 serogrouped	253 *secY* (85.7%);<br>62 *rrs* (21.0%);<br>10 *lfb1* (3.4%);<br>8 *rpoB* (2.7%);<br>5 *gyrB* (1.7%);<br>and 5 *lipL32* (1.7%).	147 *L. interrogans* (44.8%);<br>68 *L. borgpetersenii* (20.8%);<br>46 *L. santarosai* (14.0%);<br>28 *L. noguchii* (8.6%);<br>25 *L. kirschneri* (7.6%);<br>4 *L. venezuelensis* (1.2%);<br>3 *L. wolffii* (0.9%);<br>and 7 not defined (2.1%).	34.3% Sejroe;<br>65.7% non-Sejroe	218 samples from the renal tract (66.4%);<br>82 from genital samples (25.0%);<br>6 from fetal tissues (1.8%);<br>and 1 from serum (0.3%).
