# Supplementary material for: Genetic Epidemiology of Bovine Leptospirosis: A Global Perspective from Sequence and Genome Datasets
Source: Animals (Basel). 2026 Jul 2;16(13):2017. doi: 10.3390/ani16132017 (PMC13359918; doi:10.3390/ani16132017)
Supplement: Supplementary file 1 [file animals-16-02017-s001.zip › Supplementary Table S4.pdf]

Table S4: Temporal distribution of sequence and genome records of *Leptospira* spp. from bovine deposited in public datasets

	**1984-1995**	**1996-2005**	**2006-2015**	**2016-2025**
**Quantity of sequence entries**	11	11	166	372
**Type of entries (Methodology)**	Single-locus sequencing	Single-locus sequencing	Single-locus sequencing (71.1%),<br>Multi Locus Sequencing Typing (27.1%),<br>Whole Genome Sequencing (1.8%)	Single-locus sequencing (81.2%),<br>Whole Genome Seqecing (15.6%),<br>Multi Locus Sequencing Typing (2.95%)
**Genetic markers**	*rpoB* (36.3%),<br>*rrs* (36,3%),<br>*sphB* (27.3%)	*lipL32* (45.4%),<br>*rpoB* (36.4%),<br>*rrs* (9%),<br>*secY* (9%)	*secY* (29.5%),<br>*rpoB* (14.4%),<br>*flaB* (12%),<br>*rrs* (10.2%),<br>*lfb1* (4.8%)	*secY* (55.1%),<br>*rrs* (11.8%),<br>*lfb1* (9.1%),<br>*glmU* (2.4%),<br>*gyrB* (1.6%),<br>*lipL32* (1.1%)
**Species**	*L. borgpetersenii* (36.3%),<br>*L. interrogans* (36.3%),<br>*L. santarosai* (18.2%),<br>not discriminated (9%)	*L. borgpetersenii* (36.4%),<br>*L. interrogans* (18.2%),<br>*L. wolffi* (18.2%),<br>not discriminated (27.3%)	*L. interrogans* (25.3%),<br>*L. borgpetersenii* (22.3%),<br>*L. santarosai* (11.4%),<br>*L. noguchii* (6.6%),<br>*L. kirschneri* (1.8%),<br>*L. wolffii* (1.2%),<br>*L. weilli* (0.6%),<br>not discriminated (30.7%)	*L. interrogans* (39%),<br>*L. borgpetersenii* (38.2%),<br>*L. kirschneri* (7.2%),<br>*L. santarosai* (5.9%),<br>*L. noguchii* (4.6%),<br>*L. venezuelensis* (0.8%),<br>*L. wolffii* (0.3%),<br>*L. alexanderi* (0.3%), n<br>ot discriminated (3.8%)
**Geographic Region**	South America (54.6%),<br>North America (27.2%),<br>Asia (9%),<br>Europe (9%)	South America (72.7%),<br>Asia (27.3%)	South America (46.4%),<br>Asia (25.9%),<br>Africa (15.7%),<br>Europe (11.4%),<br>Oceania (0.6%).	South America (71.8%),<br>Africa (12.9%),<br>Asia (4.8%),<br>North America (4.8%),<br>Oceania (2.7%),<br>Europe (1.9%),<br>Central America (1.1%).
